# Overexpression of the CAM-Derived NAC Transcription Factor KfNAC83 Enhances Photosynthesis, Water-Deficit Tolerance, and Yield in *Arabidopsis*

**DOI:** 10.3390/cimb47090736

**Published:** 2025-09-10

**Authors:** Kumudu N. Rathnayake, Beate Wone, Madhavi A. Ariyarathne, Won C. Yim, Bernard W. M. Wone

**Affiliations:** 1 Department of Biology, University of South Dakota, Vermillion, SD 57069, USA; 2 Department of Biochemistry and Molecular Biology, University of Nevada Reno, Reno, NV 89577, USA

**Keywords:** NAC transcription factor, crassulacean acid metabolism (CAM), abiotic stress, carbon assimilation, jasmonate signaling pathway

## Abstract

Drought stress is a major constraint on plant photosynthesis, growth, and yield, particularly in the context of increasingly frequent and severe extreme weather events driven by global climate change. Enhancing photosynthetic efficiency and abiotic stress tolerance is therefore essential for sustaining crop productivity. In this study, we functionally characterized *Kalanchoë fedtschenkoi* NAC83 (*KfNAC83*), a transcription factor derived from a heat-tolerant obligate crassulacean acid metabolism (CAM) species, by constitutively overexpressing it in the C_3_ model plant *Arabidopsis thaliana*. Transgenic *Arabidopsis* lines overexpressing *KfNAC83* exhibited significantly enhanced tolerance to water-deficit and NaCl stress, along with improved photosynthetic performance, biomass accumulation, and overall productivity. Transcriptomic analysis revealed that *KfNAC83* overexpression increased key components of the jasmonate (JA) signaling pathway in both roots and shoots, suggesting a mechanistic link between *KfNAC83* activity and enhanced abiotic stress responses. Additionally, the transgenic lines displayed increased nighttime decarboxylation activity, indicative of partial CAM-like metabolic traits. These findings demonstrate that *KfNAC83* functions as a positive regulator of abiotic stress tolerance and growth, likely through modulation of jasmonate-mediated signaling and photosynthetic metabolism. This work highlights the potential of CAM-derived transcription factors for bioengineering abiotic stress-resilient crops in the face of climate change.

## 1. Introduction

Two pressing challenges confronting modern society are the growing global human population [1,2] and the increasing frequency and severity of drought events associated with global climate change [3]. Drought stress poses a significant threat to global agricultural productivity, jeopardizing the supply of food, fiber, and feed [4]. Addressing this challenge requires innovative strategies to enhance crop resilience and sustainability under environmental stress.

One promising approach involves bioengineering the crassulacean acid metabolism (CAM) pathway into C_3_ crops to improve water-use efficiency (WUE), particularly for cultivation on marginal lands [5,6,7]. However, due to the complexity of the CAM pathway, alternative strategies such as manipulating transcription factor (TF) expression offer a more tractable solution. This approach targets a small number of regulatory genes with broad downstream effects on abiotic stress-responsive networks [8,9].

Numerous studies have demonstrated the effectiveness of TF overexpression in enhancing abiotic stress tolerance. For example, overexpression of *EcbHLH57* from *Eleusine coracana* in tobacco improved tolerance to salt, oxidative, and water-deficit stress [10]; *AREB1*, an ABA-dependent TF from *Arabidopsis thaliana*, enhanced water-deficit tolerance in soybean [11]; *AtDREB1A* conferred improved water-deficit tolerance in transgenic *Indica* rice [12]; and *MYB37* increased ABA sensitivity and improved both water-deficit tolerance and seed productivity in *Arabidopsis* [13]. Additionally, members of the MYB, AP2/ERF, WRKY, NAC, and bZIP TF families have been implicated in adaptive responses to water-deficit stress [4,9,14,15].

An underexplored yet promising strategy is the identification of novel regulatory genes from extremophytes, plants adapted to extreme environments such as desiccation-tolerant species, resurrection plants, and CAM plants for use in bioengineering drought tolerance in crops [16]. CAM plants exhibit the highest WUE among all plant groups and possess exceptional tolerance to multiple abiotic stressors [17], making them ideal candidates for the discovery of abiotic stress-adaptive regulatory genes with translational potential [16]. To date, only a handful of studies have characterized such regulators from extremophytes for potential application in crop improvement [18,19,20,21].

NAC transcription factors (TFs) are plant-specific regulators known to play pivotal roles in abiotic stress responses [22,23]. However, NAC TFs from CAM species remain largely uncharacterized, despite the high abiotic stress resilience exhibited by these plants [17]. In this study, we investigated the function of *Kalanchoë fedtschenkoi* NAC83 (*KfNAC83*), a transcription factor from a heat-tolerant obligate CAM species [24,25], by constitutively overexpressing it in the C_3_ model plant *Arabidopsis thaliana*. Notably, *KfNAC83* expression was previously shown to increase sevenfold during CAM induction in older leaf pairs of *K. fedtschenkoi*, relative to the C_3_ state [16]. Our earlier work also provided preliminary evidence that *KfNAC83* enhances abiotic stress tolerance, WUE, and vegetative growth in *Arabidopsis*. These promising findings prompted a deeper investigation into the molecular mechanisms underlying these phenotypes, with the goal of identifying regulatory pathways that could be leveraged for crop improvement. Moreover, elucidating the function of *KfNAC83* in a C_3_ background might offer insights into the regulatory transitions associated with the evolution of CAM photosynthesis.

## 2. Materials and Methods

### 2.1. Sequence Alignment and Phylogenetic Analysis

Protein sequences homologous to KfNAC83 were retrieved from the Plant Transcription Factor Database (PlantTFDB v4.0; http://planttfdb.cbi.pku.edu.cn/ accessed on 12 August 2025). A total of 62 NAC transcription factor protein sequences from various plant species were used to construct a phylogenetic tree. Initially, a Neighbor-Joining (NJ) tree was generated to provide a starting topology. This was followed by a heuristic search using the Nearest-Neighbor Interchange (NNI) algorithm to explore alternative topologies and identify the one with the highest likelihood. Maximum likelihood (ML) branch lengths were computed for each candidate topology, and the tree with the greatest overall likelihood was retained. Statistical support for the resulting tree was assessed using 1000 bootstrap replicates. Evolutionary distances were estimated using the Whelan and Goldman (WAG) model in MEGA12 software [26]. Multiple sequence alignment was performed using the MUSCLE algorithm, and conserved motifs were identified and analyzed within MEGA12. Nuclear localization signal (NLS) was predicted using cNLS mapper (https://nls-mapper.iab.keio.ac.jp/cgi-bin/NLS_Mapper_form.cgi accessed on 20 August 2025).

### 2.2. Plasmid Construction

The *KfNAC83* transcription factor was cloned into the pENTR/D-TOPO entry vector via topoisomerase I mediated ligation. The resulting entry clone containing *KfNAC83* with a stop codon was recombined into the binary vector pGWB415 (CaMV35S::3xHA-attR1-attR2-NOS terminator) using Gateway^®^ LR Clonase™ II Enzyme Mix (Invitrogen, Carlsbad, CA, USA) [27]. For subcellular localization, *KfNAC83* without a stop codon was cloned into the binary vector pGWB405 (CaMV35S::attR1-attR2-sGFP-NOS terminator), which contains a C-terminal synthetic green fluorescent protein (sGFP) tag, using the same recombination system. Recombinant plasmids (35S::3xHA-*KfNAC83* and 35S::*KfNAC83*-sGFP) were transformed into *Escherichia coli* (NEB 10-beta competent cells, New England BioLabs, Ipswich, MA, USA), and plasmids were extracted using the QIAprep Spin Miniprep Kit (Qiagen, Hilden, Germany) according to the manufacturer’s instructions. All constructs were verified by Sanger sequencing (GENEWIZ, South Plainfield, NJ, USA).

### 2.3. Floral Dipping and Generation of Homozygous Transgenic Lines

Recombinant plasmids were introduced into *Agrobacterium tumefaciens* strain GV3101 using the freeze–thaw method [28]. *Arabidopsis thaliana* (Col-0) plants were transformed with 35S::3xHA-*KfNAC83* and 35S::*KfNAC83*-sGFP constructs via the floral dip method [29]. T_0_ and T_1_ seeds were screened on full-strength Murashige and Skoog (MS) medium supplemented with 50 µg/mL kanamycin in a Percival LED-30HL1 growth chamber (Percival, Perry, IA, USA) at 22 °C under a 16/8 h light/dark cycle with 120–150 μmol m^−2^ s^−1^ light intensity. T_2_ lines were selected based on a 3:1 segregation ratio for kanamycin resistance. Four independent homozygous T_3_ lines overexpressing *KfNAC83* (#6, #11, #23, and #24) were used for further experiments.

### 2.4. Subcellular Localization of 35S::KfNAC83-sGFP

Homozygous T_3_ seedlings expressing 35S::*KfNAC83*-sGFP and control seedlings expressing 35S::sGFP were used to determine subcellular localization. Ten-day-old roots and leaves were stained with Fluoroshield™ containing DAPI (Sigma-Aldrich, St. Louis, MO, USA) for 10 min (roots) or 30–45 min (leaves) at room temperature. Samples were imaged using a Nikon A1 confocal laser-scanning microscope (Nikon, Tokyo, Japan) with 60× and 40× oil immersion objectives for leaves and roots, respectively. GFP and DAPI were excited at 488 nm and 405 nm, respectively.

### 2.5. Quantitative Real-Time PCR (qRT-PCR) Analysis of OxKfNAC83 Lines

Total RNA was extracted from 100 mg of leaf, root, and stem tissues of *OxKfNAC83* lines and WT using the RNeasy Plant Mini Kit (Qiagen, Hilden, Germany). qRT-PCR was performed using the Luna^®^ Universal One-Step RT-qPCR Kit (New England Biolabs, Ipswich, MA, USA) following the manufacturer’s protocol on a QuantStudio 3 Real-Time PCR System (Applied Biosystems, Foster City, CA, USA). Expression levels of *KfNAC83* were quantified using the primers 5′-CGGCATAGACCGCAAGATTA-3′ and 5′-CAGTCCTGGTTCCCTTGTTAG-3′. Expression was normalized to the reference gene *ACT2* (*At3G18780*) using primers 5′-CTACGAGCAGGAGATGGAAAC-3′ and 5′-TCTGAATCTCTCAGCACCAATC-3′ [30], yielding amplicon sizes of 104 bp and 107 bp, respectively. Each reaction was performed with three biological replicates and three technical replicates. Relative expression was calculated using the 2^−ΔΔCT^ method [31] with QuantStudio™ Design and Analysis Software v1.5.0. Selected *OxKfNAC83* lines were used for downstream analyses.

### 2.6. Morphological Characterization of OxKfNAC83 Lines

*OxKfNAC83* lines and WT were grown in soilless–perlite potting mixture (Scott, Miracle-Gro, Marysville, OH, USA) in 89 mm square pots (0.3 L rooting volume; Kord, Inc., Toronto, ON, Canada) under a 16/8 h light/dark photoperiod for four weeks. Rosette diameter was measured using a Vernier caliper. Detached rosettes were photographed with an Olympus TG-5 camera, and rosette area was quantified using ImageJ v1.51. Leaf number per rosette was also recorded.

For biomass measurements, sterilized seeds were sown on full-strength MS medium. After stratification, plates were transferred to a Percival LED-30HL1 growth chamber at 22 °C under a 16/8 h light/dark cycle with 120–150 μmol m^−2^ s^−1^ light intensity. Fresh weight was recorded for 3-week-old seedlings; dry weight was measured after drying at 60 °C for 24 h.

### 2.7. Water-Deficit Stress Assay

To assess water-deficit tolerance, *OxKfNAC83* lines and WT were grown in soilless medium in 89 mm square pots under a 16/8 h light/dark photoperiod. After 14 days of well-watered growth, water was withheld for 15 and 20 days. Plants were then re-watered for 7 days, and survival rates were calculated based on the number of green, healthy plants. After two months of recovery, seed yield per surviving plant was measured. Experiment was repeated three times with 30 plants per genotype.

### 2.8. In Vitro Water-Deficit Assay Using PEG

Water-deficit tolerance was also evaluated in vitro using polyethylene glycol (PEG 8000) to simulate osmotic stress. Seeds of *OxKfNAC83* lines (#6, #11, #23, and #24) and WT were surface sterilized using chlorine gas [32]. Seeds were sown on half-strength MS medium supplemented with 0%, 25%, 40%, or 55% PEG, corresponding to water potentials of −0.25 MPa (control), −0.5 MPa, −0.7 MPa, and −1.2 MPa, respectively [33]. Seeds were stratified in darkness at 4 °C for 3 days, then transferred to a Percival LED-30HL1 growth chamber at 22 °C under a 16/8 h light/dark cycle with 120–150 μmol m^−2^ s^−1^ light intensity. The experiment was repeated three times with 36 seedlings per genotype. Seed germination and green cotyledon emergence percentages were recorded.

### 2.9. Seed Yield Measurement of OxKfNAC83 Lines

For seed yield analysis, *OxKfNAC83* lines and WT plants were grown under well-watered conditions in soilless medium in 89 mm square plastic pots (0.3 L rooting volume; Kord, Inc., Toronto, ON, Canada) for two months in a growth chamber under a 16/8 h light/dark photoperiod. After seed maturation, total seed weight per plant was measured for 30 plants per line. The experiment was repeated three times. Seed yield was also compared with that from plants subjected to acute water-deficit treatments.

### 2.10. Water-Use Efficiency Measurement

Water-use efficiency (WUE) was assessed using a closed system as described by Wituszynska and Karpiński [34] and Wituszynska et al. [35]. Briefly, 50 mL conical tubes were filled with a 1:1 soil-perlite mixture and 35 mL of water. Each tube was capped with a lid containing a central hole, into which 5–10 seeds were placed. Tubes were kept at 4 °C in a humid environment for 2 days, then transferred to a growth chamber under a 16/8 h light/dark photoperiod. After 5–7 days of germination, excess seedlings were removed, leaving one seedling per tube. Initial tube weight (W_0_) was recorded. After four weeks, rosettes were harvested and dried at 105 °C for 3 h to determine dry weight (DW). Final tube weight (W) was recorded, and water loss was calculated as W_0_ − W (g), assuming 1 g = 1 mL of water. WUE was calculated as DW (mg) per mL of water used.

### 2.11. In Vitro NaCl Stress Response

To assess salt stress tolerance, sterilized seeds of *OxKfNAC83* lines (#6, #11, #23, and #24) and WT were sown on full-strength MS medium supplemented with 0 (control), 100, 150, 200, or 250 mM NaCl. After 14 days, seed germination and green cotyledon emergence rates were recorded. The experiment was repeated three times with 30 seeds per line per replicate.

For post-germination salt stress assays, seedlings were germinated on full-strength MS medium for 5 days, then transferred to MS medium supplemented with 100, 150, or 200 mM NaCl for 14 days. Survival rate, fresh biomass, and dry biomass were measured for 24 seedlings per line across three replicates.

For root growth assays, five-day-old seedlings grown vertically on MS medium were transferred to MS medium supplemented with 100, 150, or 200 mM NaCl. Root elongation was measured at 3-day intervals for 12 days. After 12 days, seedlings were photographed using an Olympus TG-5 camera, and root length and lateral root number were quantified using ImageJ. Each treatment included 20 seedlings per line across three replicates. All experiments were conducted under the same growth chamber conditions described above.

### 2.12. Titratable Acidity

*OxKfNAC83* lines and WT were grown for four weeks in a Percival LED-30HL1 growth chamber (Perry, IA, USA) under a 12 h photoperiod at 120–150 μmol m^−2^ s^−1^ light intensity and 22 °C. Approximately 0.5 g of leaf tissue was collected at dawn and dusk from each line (*n* = 3 biological replicates). Samples were flash-frozen in liquid nitrogen and stored at −80 °C. Frozen tissue was ground in liquid nitrogen and extracted with 10 mL of 50% (*v*/*v*) methanol at 80 °C for 10 min. Extracts were brought to original volume with water and centrifuged at 4000× *g* for 20 min (Eppendorf 5430 R, Hamburg, Germany). Supernatants were titrated to pH 7.0 with 100 mM KOH using a calibrated pH meter. Titratable acidity was calculated as (mL KOH × 0.1 M)/g fresh weight and expressed as µmol H^+^ g^−1^ FW. Two-way ANOVA followed by Tukey’s HSE test was used to assess statistical significance between lines and time points.

### 2.13. Carbohydrate Analysis

Total soluble sugar content was quantified in *OxKfNAC83* lines and WT grown in soilless medium for four weeks under a 12 h photoperiod at 120–150 μmol m^−2^ s^−1^ light intensity and 22 °C in a Percival LED-30HL1 growth chamber. The assay was performed as described by Fox and Robyt [36] and Lim et al. [37], with modifications. Briefly, 500 mg of leaf tissue was ground in liquid nitrogen and extracted with 10 mL of 50% (*v*/*v*) methanol at 80 °C for 30 min. After centrifugation at 3000× *g* for 10 min, 50 μL of 5% (*v*/*v*) phenol was added to an equal volume of supernatant. Samples were mixed gently and placed on ice. Then, 250 μL of concentrated sulfuric acid was added, and samples were incubated at 80 °C for 30 min. Absorbance was measured at 490 nm using a Multiskan™ GO microplate spectrophotometer (Thermo Scientific™, Waltham, MA, USA).

### 2.14. Photosynthetic and Carboxylation Efficiencies

*OxKfNAC83-24* (Line #24) was selected for photosynthetic measurements due to its highest *KfNAC83* expression in leaves and stems and its use in time-series RNA-Seq analysis. Light response (A/I) curves were measured using a LI-6800 portable gas-exchange system with a fluorometer head (LI-COR Biosciences, Lincoln, NE, USA). Plants were dark-adapted for 30 min prior to measurement. Measurements were conducted at a constant CO_2_ concentration of 400 μmol mol^−1^ using red-blue actinic light (90%/10%). Assimilation rate (A) and effective quantum yield of Photosystem II (ΦPSII) were recorded simultaneously using a rectangular saturating flash (8000 μmol m^−2^ s^−1^) under stepwise irradiance (I) increments from 0 to 2000 μmol m^−2^ s^−1^ at 2 min intervals. For each plant, three leaves were measured, and the average was used per replicate. Data were fitted to nine mathematical models, and the model with the lowest sum of squared errors (Equation (2) from Kaipiainen [38] was selected as the best fit [39].

The response of net photosynthesis (A) to intercellular CO_2_ concentration (Ci) was also measured using the LI-6800 system to determine carboxylation efficiency. Measurements were conducted at a saturating photosynthetic photon flux density (PPFD) of 200 μmol m^−2^ s^−1^. CO_2_ concentration (Ca) was initially set at 400 μmol mol^−1^, then decreased stepwise to 300, 200, 100, and 50 μmol mol^−1^, followed by increases to 100, 200, 300, 400, 500, 700, 900, 1000, and 1500 μmol mol^−1^. Three leaves per plant were measured, and the average was used per replicate. CO_2_ assimilation rate and Ci were calculated using the equations of von Caemmerer and Farquhar [40]. Maximum rates of Rubisco carboxylation (Vc_max_), electron transport for RuBP regeneration (J_max_), and triose phosphate utilization (TPU) were estimated at 25 °C using equations from Sharkey et al. [41].

### 2.15. Statistical Analysis

All phenotypic and physiological data were analyzed using one-way or two-way analysis of variance (ANOVA), followed by Tukey’s honestly significant difference (HSE) test for multiple comparisons in RStudio (version 1.1383). Results are presented as mean ± standard deviation (SE) from at least three independent biological replicates. Statistical significance is indicated as *** *p* < 0.001, ** *p* < 0.01.

### 2.16. RNA Extraction for Water-Deficit and Time-Course Analyses

For water-deficit transcriptomic analysis, total RNA was extracted from 16 leaf samples (two genotypes: *OxKfNAC83-24* and WT × two treatments: well-watered and water-deficit × four biological replicates) using the RNeasy Plant Mini Kit (Qiagen, Hilden, Germany).

For time-course analysis, total RNA was extracted from 32 leaf samples (two genotypes × four time points: 09:00, 15:00, 21:00, and 03:00 h × four biological replicates) using the same kit. RNA quantity and quality were assessed using a Nanophotometer (Implen Inc., Westlake Village, CA, USA) and an Agilent 2100 Bioanalyzer (Agilent Technologies, Santa Clara, CA, USA).

### 2.17. Illumina Sequencing and Data Quality Control

All RNA samples were processed using the Illumina TruSeq RNA Sample Prep Kit for cDNA library construction, including poly(A) RNA purification, fragmentation, cDNA synthesis, and adaptor/barcode ligation. Sixteen libraries were sequenced on the Illumina NovaSeq 6000 platform (Novogene Corporation Inc., Sacramento, CA, USA) to generate >20 million 150 bp paired-end reads per sample. Quality control included trimming, adapter removal, and filtering of low-quality reads. High-quality reads were mapped to the *Arabidopsis thaliana* reference genome using HISAT2 with default parameters. Mapping results were visualized using the Integrative Genomics Viewer (IGV).

For the time-course experiment, 32 libraries from *OxKfNAC83-24* and WT across four time points were sequenced on the Illumina NovaSeq 6000 platform using 150-cycle paired-end sequencing. After filtering, >40 million paired-end reads were obtained per sample, with an average GC content of 45.91% and a sequencing error rate of 0.02%. Mapping rates ranged from 94.18% to 96.11%, with uniquely mapped reads ranging from 75.19% to 82.4%.

### 2.18. Identification of Differentially Expressed Genes (DEGs), Gene Ontology (GO), and Pathway Analysis

Normalized gene expression was calculated in fragments per kilobase of exon per million mapped reads (FPKM). Differential expression analysis was performed using DESeq2 v2_1.6.3. Genes with ≥2-fold change and FPKM ≥ 3 in at least one condition were considered differentially expressed. Volcano plots were generated to visualize DEG distributions.

Gene Ontology (GO) and Kyoto Encyclopedia of Genes and Genomes (KEGG) pathway enrichment analyses were conducted using the ClusterProfiler package to identify significantly enriched biological functions and pathways. Protein–protein interaction (PPI) enrichment analysis was performed using BioGRID (https://thebiogrid.org/ accessed on 30 July 2020). Networks containing 3–500 proteins were further analyzed using the Molecular Complex Detection (MCODE) algorithm via Metascape (https://metascape.org accessed on 30 July 2020). Resulting clusters were visualized in Cytoscape (v3.8.0).

## 3. Results

### 3.1. Phylogenetic Analysis of the KfNAC83 Gene

Phylogenetic analysis was performed using 62 NAC transcription factors (TFs) from various plant species to determine the evolutionary relationship of the KfNAC83 protein. The analysis revealed that KfNAC83 clusters closely with several known abiotic stress-responsive NAC proteins, including AtNAC35 which regulates cold stress response, OsNAC45 which is involved in drought and salt stress responses, and SlNAC2, which enhances abiotic stress tolerance through modulation of glutathione metabolism (Figure S1A). Multiple sequence alignment with characterized abiotic stress responsive NAC proteins, such as AaNAC1, AhNAC2, OsIGSNAC1, AmNAC1, BgNAC1, BnNAC1-1, CaNAC5, DgNAC1, GmNAC11 and HvNAC showed that these proteins share conserved motifs and contain a nuclear localization signal (NLS) within the A subdomain (Figure S1B).

### 3.2. KfNAC83 Localizes to the Nucleus in Root and Leaf Cells

To determine the subcellular localization of *KfNAC83*, *Arabidopsis* plants were transformed with a 35S::*KfNAC83*-sGFP construct and analyzed using confocal laser-scanning microscopy. The control 35S::sGFP protein localized to both the cytoplasm and nuclei of root and leaf cells, whereas the 35S::*KfNAC83*-sGFP fusion protein was exclusively localized to the nuclei (Figure S2).

### 3.3. KfNAC83 Is Highly Expressed in Leaf and Root Tissues

Four independent homozygous T_3_ transgenic lines overexpressing *KfNAC83* (#6, #11, #23, and #24) under the CaMV 35S promoter were generated in *Arabidopsis thaliana*. Transcript levels of *KfNAC83* were quantified in leaf, root, and stem tissues using qRT-PCR. Expression was significantly higher in both leaves and roots, with no significant difference between these two tissues (Figure S3). In contrast, expression in stems was significantly lower.

### 3.4. Overexpression of KfNAC83 Enhances Growth, Water-Deficit Tolerance, WUE, and Seed Yield

**Figure 1:**
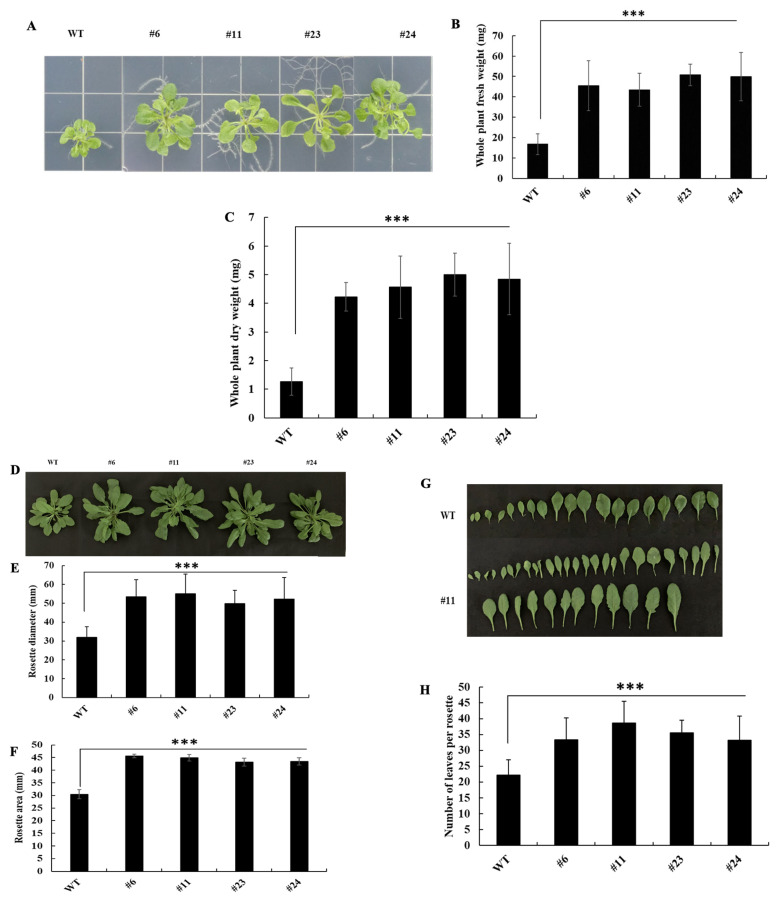
Overexpression of *KfNAC83* in *Arabidopsis* increases plant biomass. (**A**) T_2_ homozygous seeds of transgenic lines and wild type (WT) were grown on MS basal medium for three weeks. (**B**) Whole-plant fresh weight and (**C**) dry weight (n = 20) were measured after 21 days of growth. (**D**) Representative images of four-week-old, detached rosettes from transgenic lines and WT. (**E**) Rosette diameter and (**F**) rosette area were quantified using ImageJ (*n* = 20). (**G**) Representative image of detached leaves from a four-week-old rosette of *KfNAC83*-overexpressing line (#11) and WT. (**H**) Number of leaves per rosette in transgenic lines and WT (*n* = 20). Values represent means ± SE. *** *p* < 0.001. WT = wild type; #6, #11, #23, and #24 = independent transgenic lines.

To assess the impact of *KfNAC83* overexpression on growth, fresh and dry biomass were measured in *OxKfNAC83* lines and WT plants grown on MS medium for three weeks. *OxKfNAC83* lines exhibited a 2.5–3.0-fold increase in fresh biomass and a 3.3–3.9-fold increase in dry biomass compared to WT (Figure 1A–C). In four-week-old plants grown in soilless medium, rosette diameter and area were increased by 1.5–1.7-fold and 1.0–1.4-fold, respectively, in *OxKfNAC83* lines relative to WT (Figure 1D–F). Additionally, the number of leaves per rosette was 1.4–1.7-fold higher in *OxKfNAC83* lines (Figure 1G–H), indicating enhanced vegetative growth.

**Figure 2:**
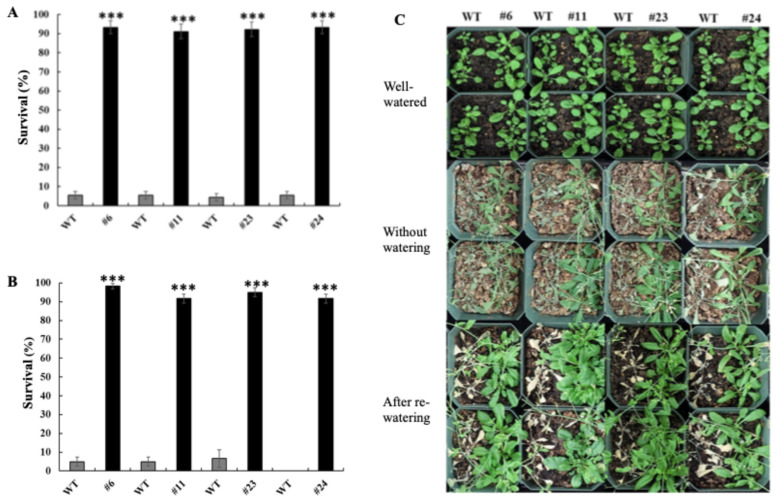
*KfNAC83*-overexpressing lines exhibit enhanced water-deficit tolerance. (**A**) Survival percentages following the 15 day water-deficit treatment. (**B**) Survival percentages following the 20 day water-deficit treatment. (**C**) Representative image from the acute water-deficit treatment, illustrating phenotypic differences between genotypes under well-watered, water-deficit (no watering), and re-watered conditions. Error bars represent ± SE from three independent experiments (*n* = 90). *** *p* < 0.001. WT = wild type; #6, #11, #23, and #24 = independent transgenic lines.

To evaluate water-deficit tolerance, *OxKfNAC83* transgenic lines and WT plants were subjected to acute water-deficit treatments lasting 15 and 20 days, following an initial 14 day period of well-watered growth. After a 7 day recovery period with re-watering, *OxKfNAC83* lines exhibited over 90% survival, whereas only 4–6% of WT plants recovered under either the 15 day (Figure 2A) or 20 day (Figure 2B,C) treatment conditions. These results demonstrate that overexpression of *KfNAC83* significantly enhances water-deficit tolerance in transgenic plants.

In vitro water-deficit tolerance was further assessed using PEG8000 to simulate low water potential. Under control conditions (0% PEG), both *OxKfNAC83* and WT seeds germinated at similar rates (Figure S4A). However, at 25% (−0.5 MPa) and 40% (−0.7 MPa) PEG, WT germination and green cotyledon emergence were significantly reduced compared to *OxKfNAC83* lines (Figure S4A,B). No germination occurred at 55% PEG. These results indicate that *OxKfNAC83* seeds exhibit enhanced tolerance to water-deficit stress during germination.

**Figure 3:**
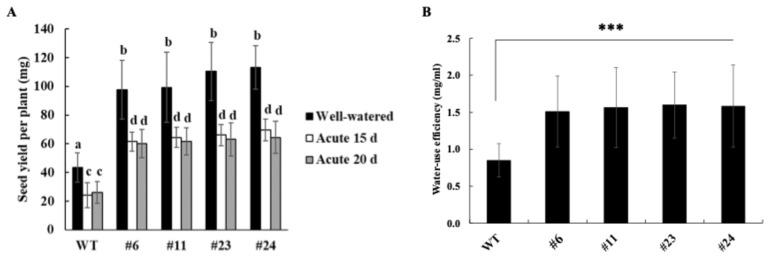
*KfNAC83* overexpression improves seed yield and water-use efficiency (WUE). (**A**) Seed yield per plant was measured under normal conditions and after 15- and 20-day water-deficit stress treatments. (**B**) WUE was calculated from water loss and shoot dry weight in four-week-old plants (*n* = 25). Error bars represent ± SE from three independent experiments. Bars with the same letters are not significantly different (*p* < 0.001); different letters indicate significant differences. *** *p* < 0.001. WT = wild type; #6, #11, #23, and #24 = independent transgenic lines.

To determine whether water-deficit tolerance affected reproductive output, seed yield per plant was measured after two months of recovery from water-deficit stress. *OxKfNAC83* lines produced significantly more seeds than WT under both water-deficit and well-watered conditions (Figure 3A), indicating that *KfNAC83* enhances water-deficit resilience without compromising productivity.

Water-use efficiency (WUE), defined as biomass produced per unit of water consumed, was also significantly higher in *OxKfNAC83* lines—by 1.7–1.8-fold compared to WT (Figure 3B).

### 3.5. Overexpression of KfNAC83 Enhances NaCl Tolerance

To evaluate whether *KfNAC83* overexpression confers salt stress tolerance, seed germination and green cotyledon emergence were assessed in *OxKfNAC83* lines and WT under increasing NaCl concentrations. Under control conditions and 100 mM NaCl, no significant differences were observed between *OxKfNAC83* lines and WT (Figure S5A–C). However, at 150, 200, and 250 mM NaCl, WT germination was significantly reduced (19–40%), while *OxKfNAC83* lines maintained high germination rates (81–93%) (Figure S5B). Similarly, green cotyledon emergence in WT was severely impaired at 150 mM NaCl and completely absent at 200 and 250 mM, whereas *OxKfNAC83* lines produced green cotyledons at 200 mM NaCl, though most became chlorotic by day 14 (<4% remained green) (Figure S5A,C). At 250 mM NaCl, none of the *OxKfNAC83* seedlings remained green after 14 days. These results indicate that *OxKfNAC83* lines exhibit enhanced salt tolerance during seed germination and early seedling development.

To assess salt tolerance at later developmental stages, five-day-old seedlings were transferred to MS medium supplemented with 100, 150, or 200 mM NaCl and grown for 14 days. On control medium, all lines showed 100% survival (Figure S6A,B). At 100 mM NaCl, survival rates were similar between *OxKfNAC83* and WT. However, at 150 mM NaCl, *OxKfNAC83* lines exhibited 93–97% survival, while WT survival dropped to 31%. At 200 mM NaCl, WT survival was <7%, whereas *OxKfNAC83* lines maintained 19–26% survival (Figure S6A,B). Fresh and dry biomass were significantly higher in *OxKfNAC83* lines than WT under all NaCl treatments (100–200 mM), while no differences were observed under control conditions (Figure S6C,D).

To further investigate root growth under salt stress, five-day-old seedlings were transferred to MS medium containing 0, 100, 150, 200, or 250 mM NaCl and grown vertically for 12 days. Root elongation was measured every 3 days. No significant differences were observed under control conditions, but root elongation was significantly less inhibited in *OxKfNAC83* lines than in WT at all NaCl concentrations (Figure S7A–E). Additionally, *OxKfNAC83* lines developed significantly more lateral roots than WT under control, 100 mM, and 150 mM NaCl conditions. At 200 mM NaCl, lateral root numbers were similar between genotypes (Figure S7F).

### 3.6. KfNAC83 Overexpression Increases Organic Acid and Carbohydrate Accumulation

**Figure 4:**
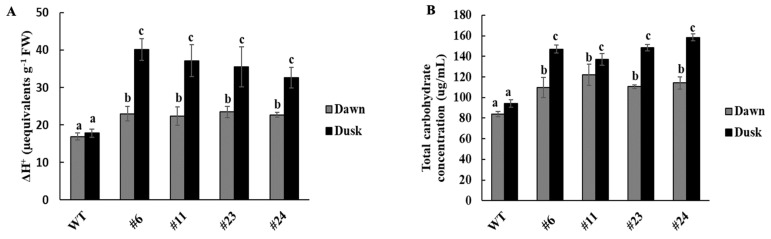
*OxKfNAC83* lines accumulate more organic acids and carbohydrates. T_2_ homozygous seeds of *OxKfNAC83* lines and WT were grown on soilless medium for four weeks under a 12 h photoperiod. (**A**) Titratable acidity was measured to quantify organic acid content (*n* = 3). (**B**) Total soluble sugar concentration was measured at dawn and dusk (*n* = 3). Values represent means ± SE. Bars with the same letters are not significantly different (*p* < 0.001); different letters indicate significant differences. WT = wild type; #6, #11, #23, and #24 = independent transgenic lines.

To determine whether *KfNAC83* overexpression induces CAM-like metabolic traits, titratable acidity (TA) and carbohydrate content were measured in *OxKfNAC83* lines and WT. All *OxKfNAC83* lines exhibited a significant 1.8–2.2-fold increase in nighttime organic acid accumulation compared to WT (Figure 4A). Conversely, daytime organic acid levels were significantly reduced (1.4–1.7-fold) in *OxKfNAC83* lines relative to nighttime levels (Figure 4A). In WT, no significant diel fluctuation in organic acid content was observed. Total carbohydrate content was significantly higher in *OxKfNAC83* lines than in WT at both daytime and nighttime (Figure 4B). Moreover, *OxKfNAC83* lines showed a significant diel difference in carbohydrate levels, which was not observed in WT.

### 3.7. KfNAC83 Enhances Photosynthetic and Carboxylation Efficiencies

**Figure 5:**
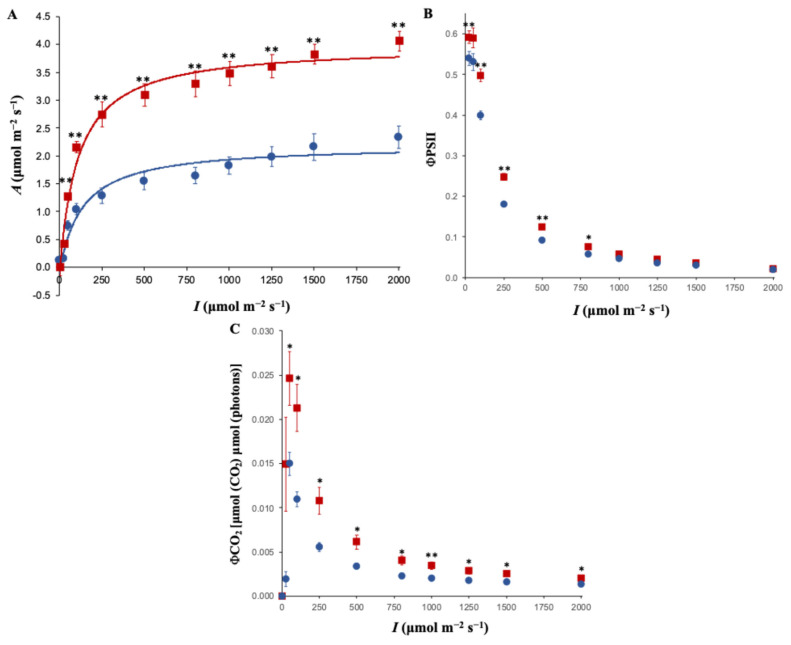
Net photosynthetic light-response (A/I) analysis of *OxKfNAC83* and WT plants. (**A**) Net CO_2_ assimilation rate (*A*), (**B**) Quantum yield of Photosystem II (ΦPSII), and (**C**) Quantum yield of CO_2_ (ΦCO_2_) were measured across a range of irradiance (*I*). Data are from T_2_ progeny of line #24 and WT (*n* = 3 per treatment). Solid lines: red = *OxKfNAC83,* blue = WT. Symbols: red squares = OxKfNAC83, blue circles = WT. Error bars represent standard error of the mean. * *p* < 0.05, ** *p* < 0.01.

To assess real-time photosynthetic performance, light response (A/I) curves were measured at a constant CO_2_ concentration of 400 μmol mol^−1^. *OxKfNAC83-24* plants exhibited significantly higher CO_2_ assimilation rates than WT at irradiances above 50 μmol m^−2^ s^−1^ (Figure 5A). The initial slope of the A/I curve (quantum efficiency) was significantly steeper in *OxKfNAC83* plants (0.019 ± 0.001) compared to WT (0.009 ± 0.003; *p* = 0.006). Maximum photosynthetic rate (A_max_) was also significantly higher in *OxKfNAC83* plants (3.7 ± 0.20 μmol CO_2_ m^−2^ s^−1^) than in WT (2.1 ± 0.41 μmol CO_2_ m^−2^ s^−1^; *p* = 0.005).

Quantum yield of PSII (ΦPSII) was significantly higher in *OxKfNAC83* plants across irradiances from 25 to 800 μmol m^−2^ s^−1^ (Figure 5B), indicating more efficient light utilization. Estimated quantum yield of CO_2_ at 200 μmol m^−2^ s^−1^ was also significantly higher in *OxKfNAC83* plants (0.010 ± 0.00072) compared to WT (0.006 ± 0.00105; *p* = 0.004) (Figure 5C).

**Figure 6:**
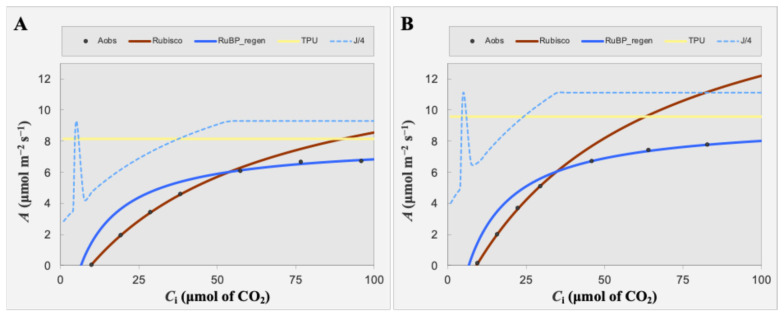
CO_2_-response (A/Ci) analysis of *OxKfNAC83* and WT plants. (**A**) WT and (**B**) *OxKfNAC83* plants were analyzed for net CO_2_ assimilation rate (**A**) in response to intercellular CO_2_ concentration (Ci). Data are from T_2_ progeny of line #24 and WT (*n* = 3 per treatment). Black circles represent observed *A* values.

Carboxylation efficiency was assessed by measuring the response of net photosynthesis (*A*) to intercellular CO_2_ concentration (Ci) under saturating light (200 μmol m^−2^ s^−1^). *OxKfNAC83* plants exhibited significantly higher values for Vc_max_ (26 vs. 19 μmol m^−2^ s^−1^; *p* = 0.03), J_max_ (50 vs. 42 μmol m^−2^ s^−1^; *p* = 0.03), and TPU (3.6 vs. 3.1 μmol m^−2^ s^−1^; *p* = 0.04) compared to WT (Figure 6A,B).

### 3.8. Enhanced Water-Deficit Tolerance Involves Extensive Transcriptional Reprogramming

**Figure 7:**
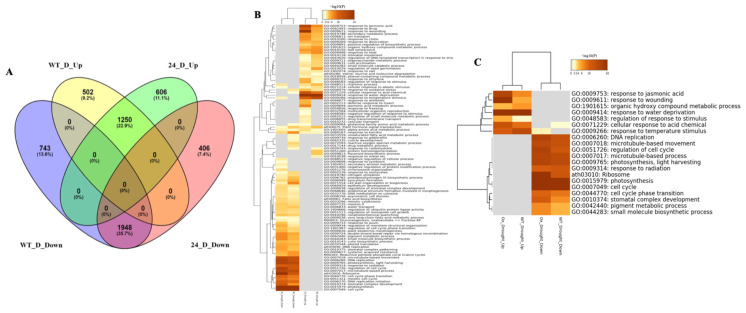
Differentially expressed genes (DEGs) and gene ontology (GO) enrichment in a *OxKfNAC83* line. (**A**) Venn diagram showing increased and decreased DEGs in *OxKfNAC83* line #24 compared to WT. (**B**) Top 100 and (**C**) top 20 enriched GO terms among DEGs, categorized by biological process, presented in hierarchical clusters.

To investigate the transcriptional basis underlying the enhanced water-deficit tolerance in *OxKfNAC83* plants, Illumina-based RNA-Seq was performed on leaf tissues. A total of 5455 differentially expressed genes (DEGs) were identified in water-deficit-treated samples compared to controls. Of these, 1012 and 1245 DEGs were unique to *OxKfNAC83-24* (L24) and WT, respectively, while 3198 DEGs were shared between both genotypes. In L24 under water-deficit conditions, 606 genes (11.1%) were significantly increased, and 406 genes (7.4%) were decreased (Figure 6). In WT, 502 genes (9.2%) were increased, and 743 genes (13.6%) were decreased (Figure 6). Among the 3198 shared DEGs, 1250 (22.9%) were increased and 1948 (35.7%) were decreased (Figure 7).

Gene Ontology (GO) enrichment analysis was performed on the DEGs using a false discovery rate (FDR) threshold of <0.005. The top 100 and top 20 enriched GO terms were hierarchically clustered based on Kappa statistics (K = 0.3) (Figure 7B). The top 20 GO terms included “response to jasmonic acid (JA),” “response to water deprivation,” “regulation of response to stimulus,” and “cellular response to acid chemicals” (Figure 7C).

GO enrichment was also applied to the protein–protein interaction (PPI) network and its MCODE-derived modules to assign biological functions. In L24 under water-deficit stress, five major network hubs were identified: JA-mediated signaling, plant hormone signal tranSEuction, intracellular signal tranSEuction, response to wounding, and response to abscisic acid (ABA) (Figure S8A). One hub included highly expressed jasmonate signaling genes such as *JAZ1*, *JAZ6*, *JAZ8*, *JAZ10*, *TIFY7*, and *TIFY10B*. ABA-responsive genes including *MYC2*, *JAZ5*, *JAZ11*, and *NAC032* were also increased in L24. Notably, Clade A protein phosphatase 2Cs (PP2Cs), including *HAI1*, a key regulator of ABA signaling, were highly expressed in L24 (Figure S8A).

In contrast, WT plants under water-deficit stress showed four major network hubs: JA-mediated signaling, response to water deprivation, response to ABA, and chlorophyll metabolic processes (Figure S8B). Although both genotypes shared JA and ABA-related hubs, the expression levels of associated DEGs were significantly higher in L24. Both L24 and WT also shared decreased hubs related to auxin-activated signaling, cell cycle regulation, and water transport (Figure S8C,D). Additionally, L24 uniquely exhibited decreased expression of seed germination-related genes such as *PDF2*, *ATML1*, and *RGL2* (Figure S8C).

### 3.9. Diel Shifts in Transcript Abundance Reveal Enhanced Photosynthetic and CAM-like Functional Associations

To explore diel transcriptional dynamics associated with enhanced photosynthesis and growth, RNA-Seq was performed on leaf tissues collected at four time points: 03:00, 09:00, 15:00, and 21:00 h. Volcano plots revealed that L24 samples had 840, 2511, 1713, and 1161 increased DEGs at 03:00, 09:00, 15:00, and 21:00 h, respectively (Figure S9A–D). Corresponding decreased DEGs were 1058, 3061, 1897, and 1102, respectively (Figure S10A–D). Within L24, 9528 DEGs were identified between 03:00 and 15:00 h, with 4717 increased and 4811 decreased genes (Figure S9E). At 09:00 and 21:00 h, 5689 and 5594 DEGs were increased and decreased, respectively (Figure S9F). Among these DEGs, core CAM-related genes such as *PEPCK* and *BCA* were significantly increased in L24 compared to WT (Figure S10A,B). However, other CAM genes including *PPC*, *PPCK*, *PPDK*, and *ALMT* did not show significant differences (Figure S10C–F).

**Figure 8:**
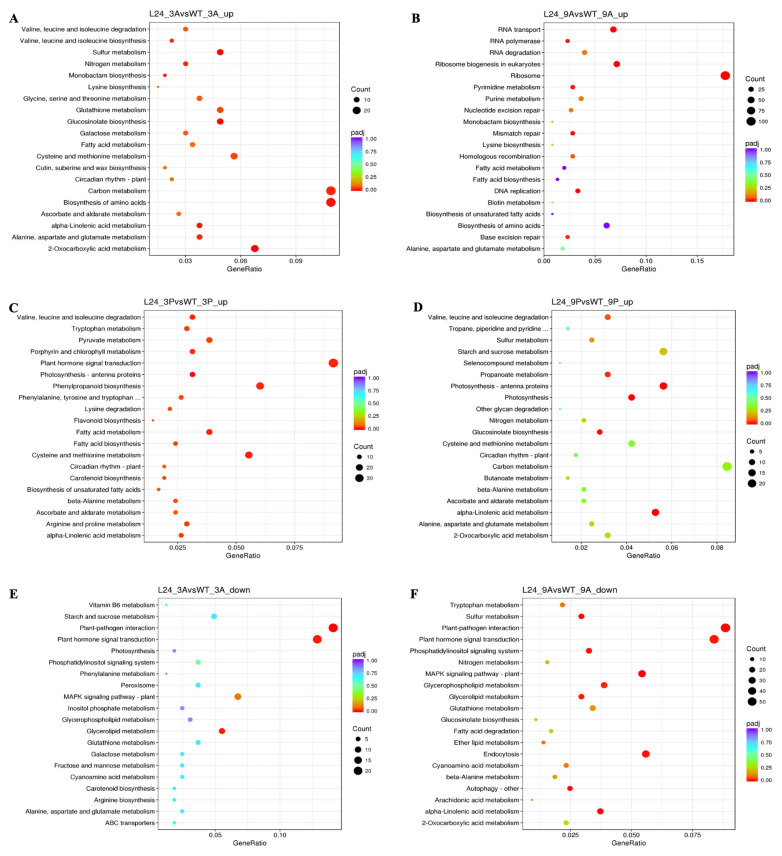

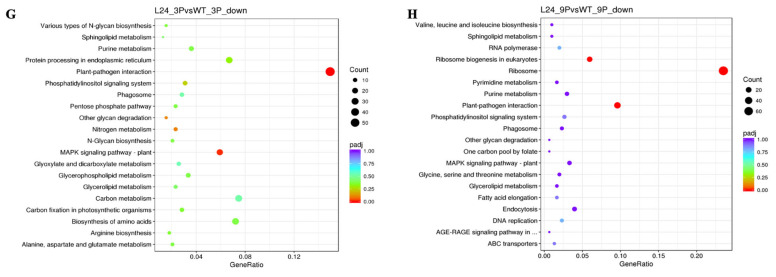
KEGG pathway enrichment analysis of DEGs in a *OxKfNAC83* line. KEGG pathway enrichment scatter plots for significantly increased genes in (**A**) L24_3A vs. WT_3A, (**B**) L24_9A vs. WT_9A, (**C**) L24_3P vs. WT_3P, and (**D**) L24_9P vs. WT_9P; and for decreased genes in (**E**) L24_3A vs. WT_3A, (**F**) L24_9A vs. WT_9A, (**G**) L24_3P vs. WT_3P, and (**H**) L24_9P vs. WT_9P. The x-axis indicates the rich factor; the y-axis lists KEGG pathway names. Dot size represents the number of genes; dot color indicates q-value. Top 20 enriched pathways are shown.

KEGG pathway enrichment analysis was performed to further characterize diel DEGs (Figure 8A–H). At 03:00 h, L24 showed enrichment in carbon metabolism and 2-oxocarboxylic acid metabolism, suggesting enhanced nocturnal CO_2_ fixation (Figure 8A). Additional enriched pathways included alanine, aspartate, and glutamate metabolism, glucosinolate biosynthesis, and glutathione metabolism. At 09:00 h, increased DEGs were associated with ribosome biogenesis, RNA transport, and DNA replication (Figure 8B). At 15:00 h, DEGs were enriched in abiotic stress-related pathways including plant hormone signal tranSEuction, arginine and proline metabolism, carotenoid and flavonoid biosynthesis, phenylpropanoid biosynthesis, and photosynthesis-antenna proteins (Figure 8C). At 21:00 h, DEGs were enriched in photosynthesis and antenna protein pathways (Figure 8D).

Conversely, carbon metabolism genes were significantly decreased at 15:00 h in L24 (Figure 8G). Plant hormone signaling and plant–pathogen interaction pathways were decreased at 03:00 and 09:00 h (Figure 8E,F). Additional abiotic stress-related pathways, including MAPK signaling, α-linolenic acid metabolism, and lipid metabolism, were also decreased at 09:00 h (Figure 8F).

### 3.10. Protein–Protein Interaction Enrichment Analysis During Diel Shifts Reveals Plant Defense and Growth Functions

To explore the functional significance of transcriptional reprogramming across diel time points, protein–protein interaction (PPI) networks were constructed for DEGs in *OxKfNAC83-24* (L24) and WT using the BioGRID database [42]. DEGs at each time point were mapped to the global *Arabidopsis* protein association network, and the MCODE algorithm [43] was applied to identify densely connected network modules.

Interestingly, clusters of significantly increased genes at all time points, except L24 09:00 were enriched in regulatory interactions associated with jasmonic acid (JA) signaling, auxin-activated signaling, and plant hormone signal tranSEuction pathways (Figure S11A–D). In the L24 03:00 sample, one cluster included *PAP2*, *IAA16*, *IAA18*, and *AXR3*, all associated with the auxin-activated signaling pathway (Figure S11A). A second cluster at the same time point included *JAZ3*, *JAZ10*, *TIFY7*, and *TIFY10B*, key components of the JA signaling pathway (Figure S11A).

In contrast, the L24 09:00 sample revealed a distinct network structure. One cluster included *SPA3*, *SPA4*, *COP1*, and *DDB1B*, genes associated with cytidine-to-uridine RNA editing. Another cluster included *CKS2*, *CKS*, *CDKD1*, and *CYCD2*, which are involved in cell cycle regulation (Figure S11B).

At 15:00, a prominent auxin-related cluster was identified, consisting of *IAA14*, *IAA16*, *IAA5*, *ARF6*, *ARF8*, *PAP2*, and *AXR3* (Figure S11C). Previous studies have shown that *IAA14* regulates lateral root development [44], while *IAA16* influences stem elongation and shoot apical dominance [45]. The auxin response factors *ARF6* and *ARF8* are known to promote JA biosynthesis and floral maturation [46].

## 4. Discussion

Collectively, our study demonstrates that overexpression of the CAM-derived transcription factor *KfNAC83* in *Arabidopsis* enhances carbon assimilation, abiotic stress tolerance, growth, and productivity. While NAC proteins are well known to regulate plant development and responses to abiotic stress [47,48], our findings show that ectopic expression of *KfNAC83*, derived from a heat-tolerant obligate CAM species, not only improves water-deficit and NaCl tolerance but also enhances photosynthetic performance and yield in a C_3_ model plant.

*KfNAC83* is a stress-responsive NAC (SNAC) transcription factor. The NAC superfamily is one of the largest transcription factor families in plants, comprising over 100 members involved in the regulation of both biotic and abiotic stress responses [22,49]. Members specifically associated with abiotic stress responses are classified as SNACs. Phylogenetic analysis revealed that *KfNAC83* is closely related to other well-characterized SNACs from species such as *Arabidopsis thaliana*, *Oryza sativa*, and *Solanum lycopersicum* (Figure S1A). Overexpression of SNAC genes such as *OsNAC2/6* and *OsNAC10* has been shown to enhance tolerance to water-deficit and salt stress [49,50]. Similarly, *ANAC019* and *ANAC055* from *Arabidopsis* are implicated in both abscisic acid (ABA)- and jasmonic acid (JA)-mediated signaling pathways [49,51]. Like other SNACs, KfNAC83 contains a putative nuclear localization signal within the A subdomain (Figure S1B). Consistent with this, *KfNAC83* expression was detected exclusively in the nuclei of root and leaf cells (Figure S2), confirming its nuclear localization. Moreover, *KfNAC83* transcript levels were elevated in both leaves and roots of transgenic lines, although no significant difference was observed between these tissues (Figure S2). Collectively, these findings suggest that *KfNAC83* plays a critical role in enhancing water-deficit stress tolerance in *Arabidopsis*, likely by increasing downstream abiotic stress-responsive genes in both leaves and roots, with a specific influence on the JA signaling pathway.

Functionally, *OxKfNAC83* plants exhibited enhanced water-deficit tolerance, surviving up to 20 days under water-deficit conditions, whereas WT plants did not (Figure 2). Transcriptomic analysis of *OxKfNAC83* plants under water-deficit stress revealed the activation of abiotic stress-responsive genes associated with the JA signaling pathway (Figure S8A). Abiotic stress is known to induce JA biosynthesis and accumulation, which in turn triggers the ubiquitin-mediated degradation of JASMONATE ZIM-DOMAIN (JAZ) proteins. This degradation relieves repression on *MYC2*, leading to the activation of JA-responsive gene expression [52,53]. JA-responsive genes are primarily involved in regulating plant defense mechanisms, including reactive oxygen species (ROS) scavenging and the production of osmoprotectants [53]. JA biosynthesis is regulated by several transcription factors (TFs), such as *MYC2*, *JAZ*, and *WRKY* [49,54]. However, the role of *NAC* TFs in JA biosynthesis and signaling under water-deficit conditions remains relatively unexplored [49,55].

Jasmonates and their derivatives are known to interact synergistically or antagonistically with other phytohormones, including auxin, ethylene, abscisic acid (ABA), salicylic acid, brassinosteroids, and gibberellins, to regulate plant growth, development, and responses to abiotic stresses such as cold, drought, salinity, heavy metals, and light [54,56,57]. Within this complex hormonal network, JA functions as a central signaling molecule [58], and its activity appears to be modulated by abiotic stress-responsive *NAC* TFs [59,60,61,62,63].

Although WT plants showed some induction of *JAZ* and *MYC2* expression under water-deficit stress, only *OxKfNAC83* plants survived prolonged water-deficit conditions. This suggests that *KfNAC83* enhances water-deficit tolerance by regulating the expression of downstream genes involved in abiotic stress adaptation. In addition to JA-related genes, several ABA signaling components, including the ABA-inducible *bHLH*-type transcription factor (*AIB*), *MYC2*, *PP2CA*, and *HAI1*, were differentially expressed in water deficit stressed *OxKfNAC83* plants (Figure S8A). *AIB* is known to play a positive role in ABA signaling in *Arabidopsis*, and *MYC2* also functions as a transcriptional activator in the ABA signaling pathway [64]. These findings suggest that the improved abiotic stress resistance observed in *OxKfNAC83* plants is mediated through transcriptional regulation of both JA and ABA signaling pathways.

Another key observation is the enhanced nighttime carbohydrate availability in *OxKfNAC83* plants, likely due to increased organic acid accumulation and subsequent decarboxylation via the PEP carboxykinase (PEPCK) pathway (Figure 4A and Figure S10A). This metabolic shift supports greater nighttime energy reserves, contributing to improved growth and productivity [65,66]. Diel fluctuations in carbohydrate and organic acid levels are hallmark features of CAM-cycling plants, where carbohydrate accumulation typically occurs during the day [67]. Interestingly, C_4_ plants such as *Saccharum* spp. also utilize the PEPCK pathway under water-limited conditions [68], suggesting a convergent mechanism for abiotic stress adaptation. Our study further showed that *KfNAC83* overexpression not only modulated the PEPCK pathway but also enhanced abiotic stress signaling, indicating a dual role in metabolic and hormonal regulation.

These findings are consistent with recent insights from Prusty and Sahoo [69], who reviewed the role of PEPCK in enhancing photosynthetic efficiency and salinity tolerance in rice. Their work highlights PEPCK as a central cytosolic decarboxylase involved in gluconeogenesis, malate metabolism, and the TCA cycle, with additional roles in regulating stomatal conductance, carbon assimilation, and energy metabolism under abiotic stress. They also emphasize PEPCK’s contribution to osmotic adjustment, ROS detoxification, and sugar accumulation, mechanisms that align with the physiological and transcriptomic responses observed in *OxKfNAC83* plants. The increased expression of *PEPCK* and associated CAM-like traits in our transgenic lines suggests that *KfNAC83* might regulate or interact with PEPCK-mediated pathways to confer enhanced photosynthetic capacity and abiotic stress resilience, mediated by the JA pathway [70]. The dual role of *KfNAC83* in regulating the PEPCK pathway and abiotic stress-responsive signaling suggests that CAM plants likely have evolved through diel transcriptional rewiring of C_3_ regulatory networks [71,72,73]. These results underscore the potential of CAM-derived transcription factors to reprogram C_3_ metabolism toward more efficient, abiotic stress-adaptive states, with implications for bioengineering climate-resilient crops.

Future studies should explore the role of *KfNAC83* and its downstream targets in other C_3_ species to assess its broader applicability in improving water-deficit tolerance, nighttime starch metabolism, and agricultural productivity. In particular, it would be valuable to investigate whether the dual role of *KfNAC83* in regulating both the PEPCK pathway and abiotic stress-responsive signaling reflects a broader mechanism by which CAM plants evolved through diel transcriptional rewiring of ancestral C_3_ regulatory networks.

## 5. Conclusions

To our knowledge, this is the first study to functionally characterize an NAC83 transcription factor from the heat-tolerant obligate CAM species *Kalanchoë fedtschenkoi*. Our findings demonstrate that overexpression of KfNAC83 in *Arabidopsis* significantly enhances tolerance to water-deficit and NaCl stress, while also improving carbon assimilation, growth, and seed yield. These phenotypic improvements are underpinned by transcriptional reprogramming of key hormonal pathways, particularly JA and ABA signaling, as well as diel regulation of photosynthetic and metabolic genes.

Importantly, *KfNAC83* overexpression also induced CAM-like traits, including nighttime organic acid accumulation and activation of the PEPCK pathway. These features are consistent with recent findings in rice, where PEPCK has been shown to enhance photosynthetic efficiency and confer salinity tolerance through its roles in gluconeogenesis, malate metabolism, and stomatal regulation [69]. The convergence of these metabolic and regulatory functions suggests that *KfNAC83* might act upstream of or in coordination with PEPCK-mediated processes to promote abiotic stress resilience and metabolic flexibility. Collectively, our results highlight the potential of CAM-derived transcription factors such as *KfNAC83* to reprogram C_3_ plant metabolism toward more efficient, abiotic stress-adaptive states, offering a promising strategy for bioengineering climate-resilient crops.

## Data Availability

Data will be made available on request.

## Supplementary Materials

The following supporting information can be downloaded at https://www.mdpi.com/article/10.3390/cimb47090736/s1.
