# Supplementary material for: Overexpression of the CAM-Derived NAC Transcription Factor KfNAC83 Enhances Photosynthesis, Water-Deficit Tolerance, and Yield in Arabidopsis
Source: Curr Issues Mol Biol. 2025 Sep 10;47(9):736. doi: 10.3390/cimb47090736 (PMC12468989; doi:10.3390/cimb47090736)
Supplement: Supplementary file 1 [file cimb-47-00736-s001.zip › cimb-3778985-supplementary.pdf]

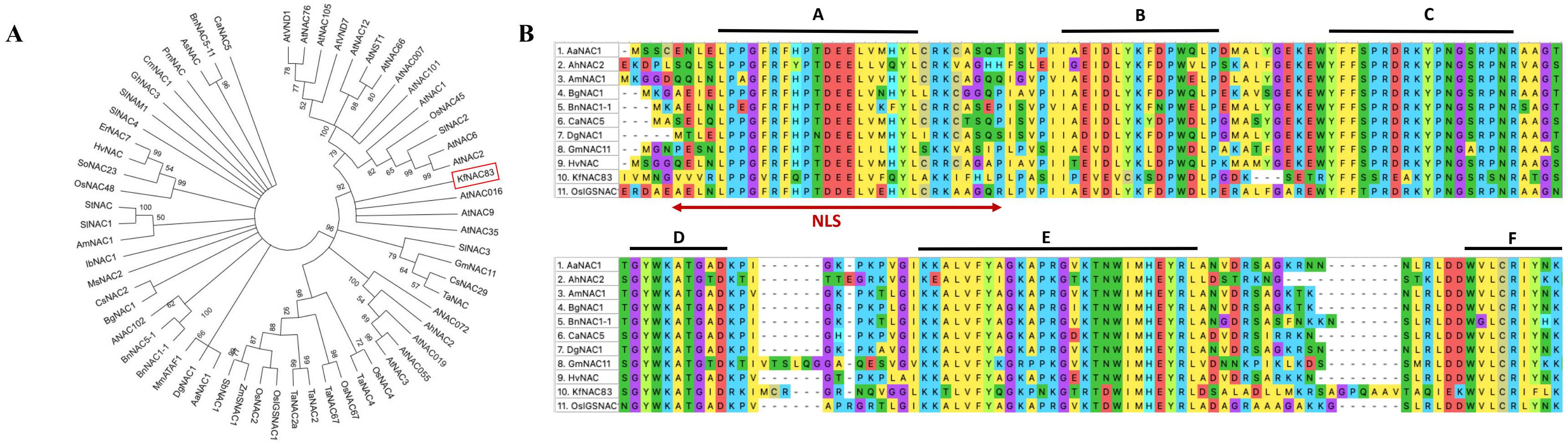

Figure S1. Phylogenetic analysis of the KfNAC83 protein. (**A**) Phylogenetic tree showing evolutionary relationships of KfNAC83 and other NAC proteins. The tree was constructed using the Maximum Likelihood (ML) method in MEGA12 with 1,000 bootstrap replicates. Numbers next to branches indicate bootstrap support values (percentage of replicate trees in which the associated taxa clustered together). The evolutionary distances were computed using the Whelan and Goldman (WAG) model with a discrete Gamma distribution across five categories to account for rate variation among sites. The analysis included 62 NAC protein sequences from various plant species. (**B**) Multiple sequence alignment showing conserved motifs (subdomains A, B, C, D, E, and F) and the predicted NLS. The cNLS Mapper web tool predicted the NLS of the KfNAC83 protein as VVRLPPGVRFQPTDEELVFQYLAKKIFHLP.

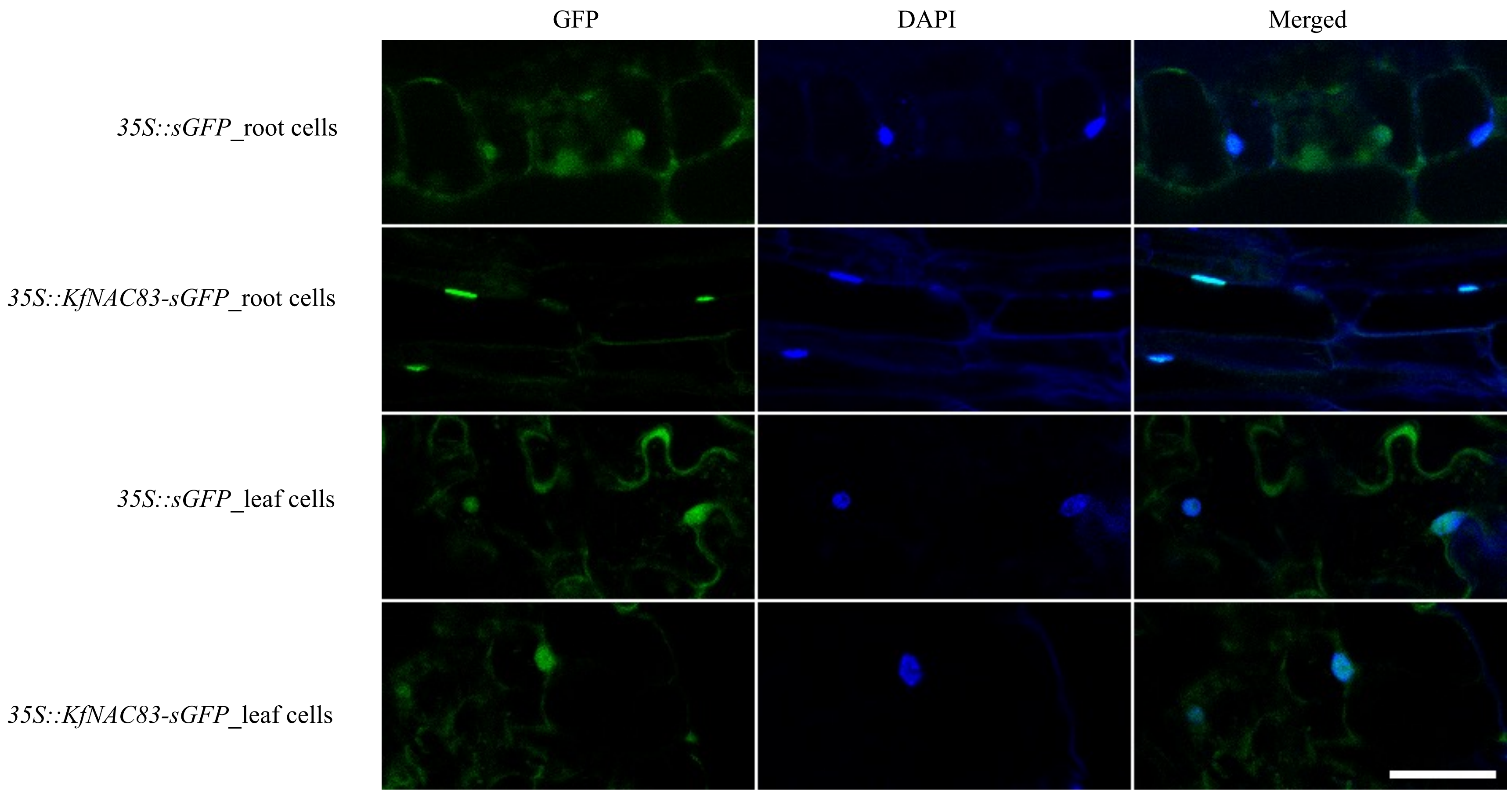

Figure S2. Subcellular localization of KfNAC83 in *Arabidopsis thaliana*. The 35S::KfNAC83-sGFP and 35S::sGFP constructs were introduced into *Arabidopsis via Agrobacterium*-mediated floral dip. Ten-day-old homozygous T_3 seedlings were examined under a confocal laser scanning microscope. White Scale bar = 50 µm.

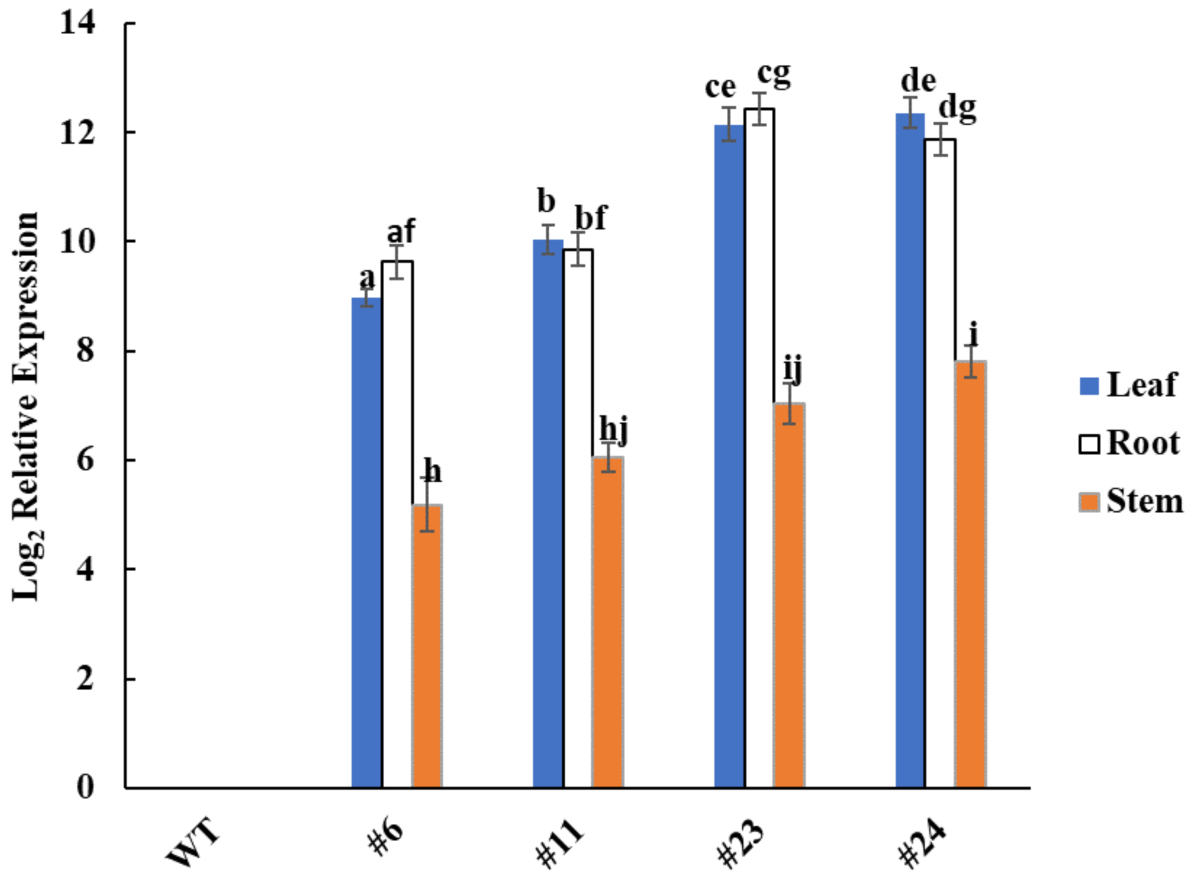

Figure S3. Relative mRNA expression levels of *KfNAC83* in different *Arabidopsis* tissues. *KfNAC83* transcript levels were quantified by qRT-PCR in leaf, root, and stem tissues of four-week-old *Arabidopsis* plants. Expression was normalized to the *ACT2* reference gene. Error bars represent ± SE from three independent experiments. Bars with the same letters are not significantly different ($P < 0.001$); different letters indicate significant differences. WT = wild type; #6, #11, #23, and #24 = independent transgenic lines.

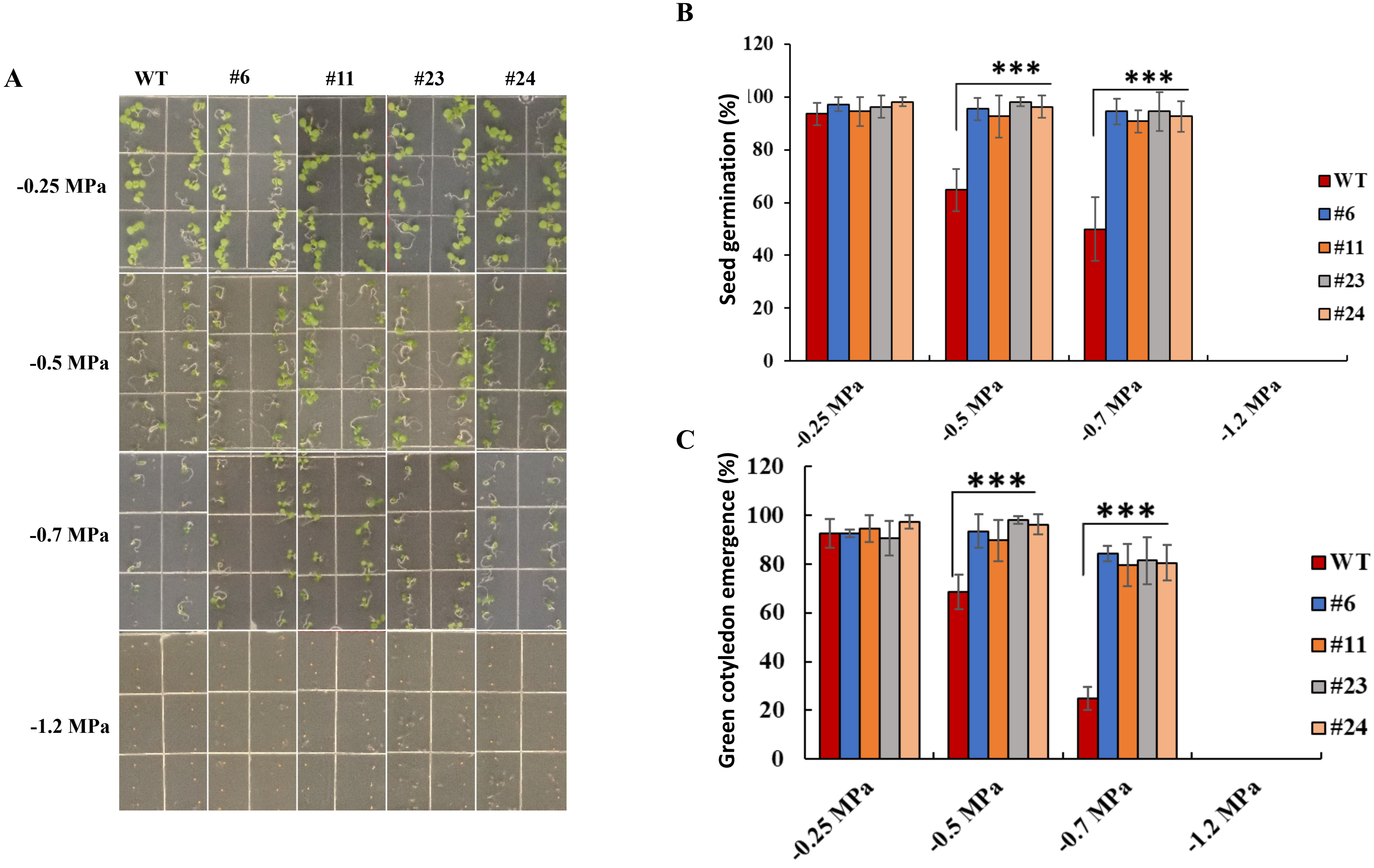

Figure S4. Overexpression of *KfNAC83* improves seed germination and cotyledon emergence under water-limited conditions. (**A**) T_2 homozygous seeds of transgenic lines and WT were exposed to PEG concentrations of 0%, 25%, 40%, and 55%, corresponding to water potentials of −0.25 MPa, −0.5 MPa, −0.7 MPa, and −1.2 MPa, respectively. (**B**) Seed germination percentage and (**C**) Green

cotyledon emergence percentage were recorded after 14 days. Error bars represent ± SE from three independent experiments. ***$P < 0.001$. WT = wild type; #6, #11, #23, and #24 = independent transgenic lines.

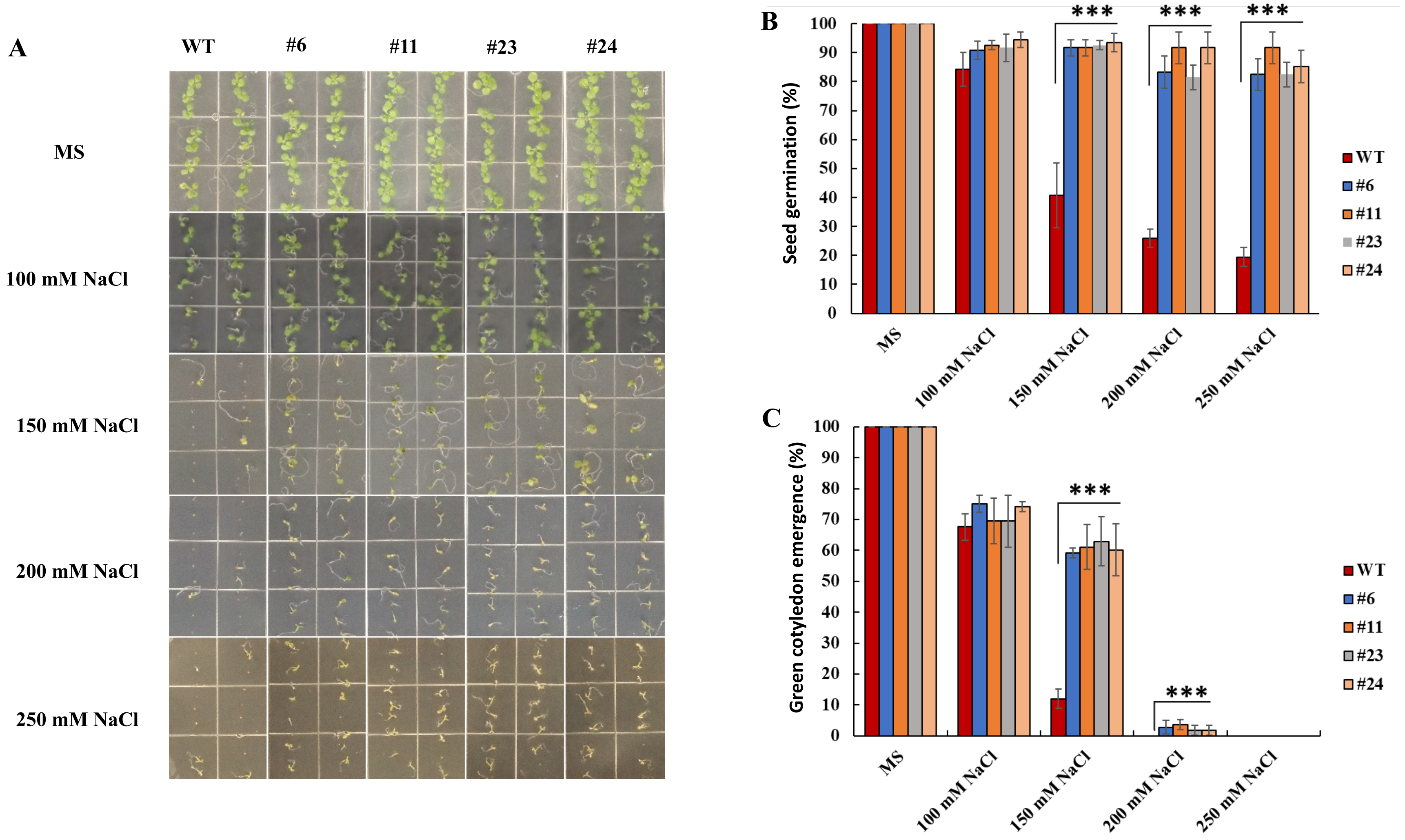

Figure S5. Overexpression of *KfNAC83* enhances seed germination and cotyledon emergence under NaCl stress. (**A**) Seeds of transgenic lines and WT were treated with 100, 150, 200, and 250 mM NaCl. (B) Seed germination percentage and (**C**) Green cotyledon emergence percentage were assessed after 14 days. Error bars represent ± SE from three independent experiments. ***P < 0.001. WT = wild type; #6, #11, #23, and #24 = independent transgenic lines.

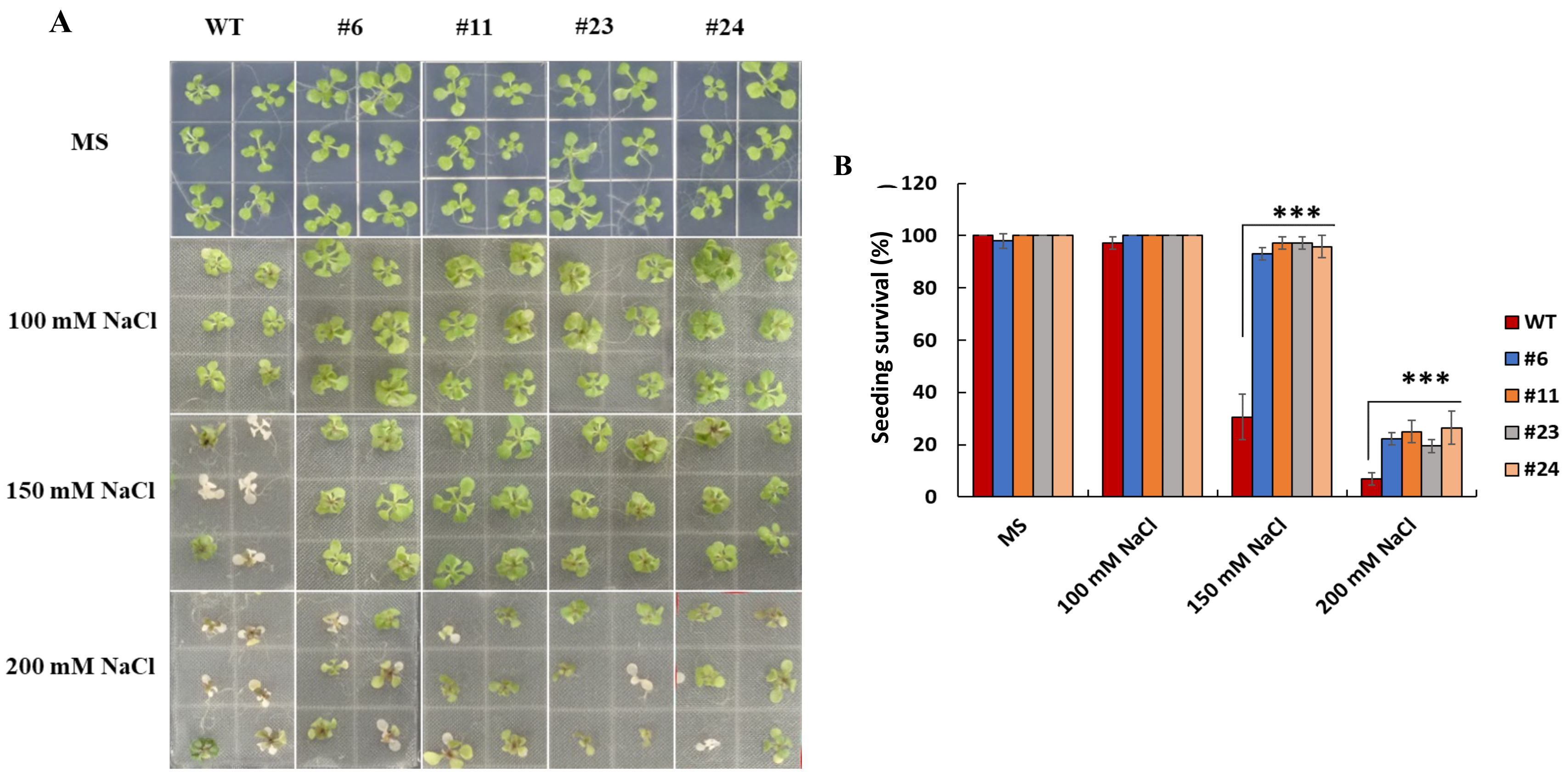
A
WT
#6
#11
#23
#24
MS
100 mM NaCl
150 mM NaCl
200 mM NaCl
B
Seeding survival (%)
120
100
80
60
40
20
0
***
***
WT
#6
#11
#23
#24
MS
100 mM NaCl
150 mM NaCl
200 mM NaCl

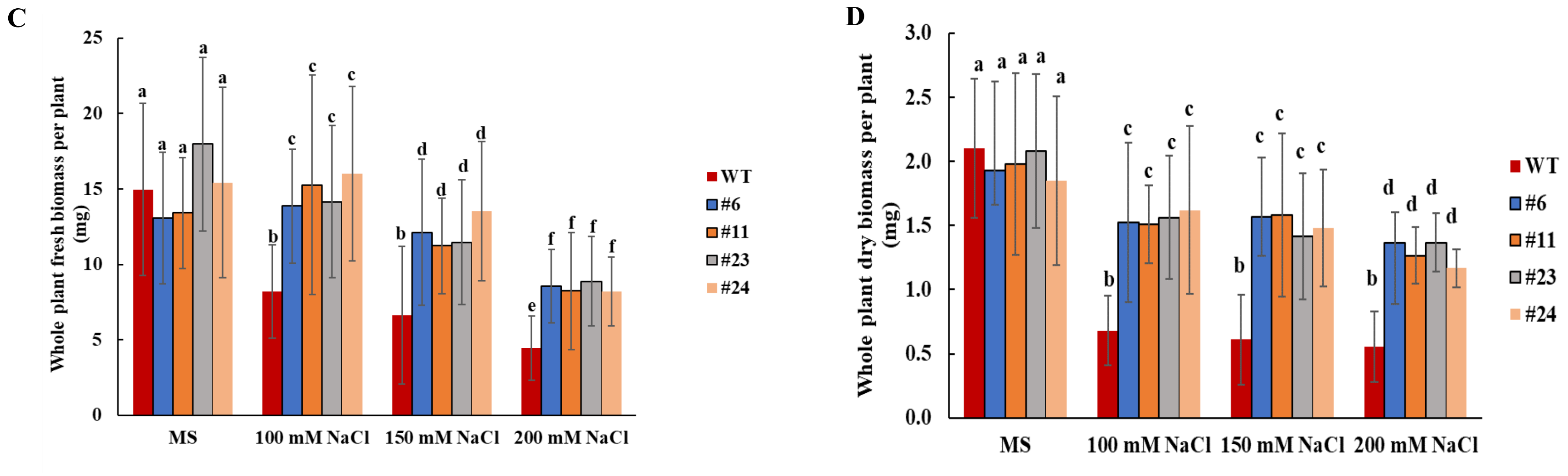

Figure S6. Overexpression of *KfNAC83* improves seedling survival under salt stress. (**A**) Five-day-old seedlings of transgenic lines and WT were treated with 100, 150, and 200 mM NaCl for 14 days. (**B**) Seedling survival percentage, (**C**) Fresh biomass, and (**D**) Dry biomass were measured. Error bars represent ± SE from three independent experiments. ***P < 0.001. Bars with the same letters are not significantly different (P < 0.001); different letters indicate significant differences. WT = wild type; #6, #11, #23, and #24 = independent transgenic lines.

A

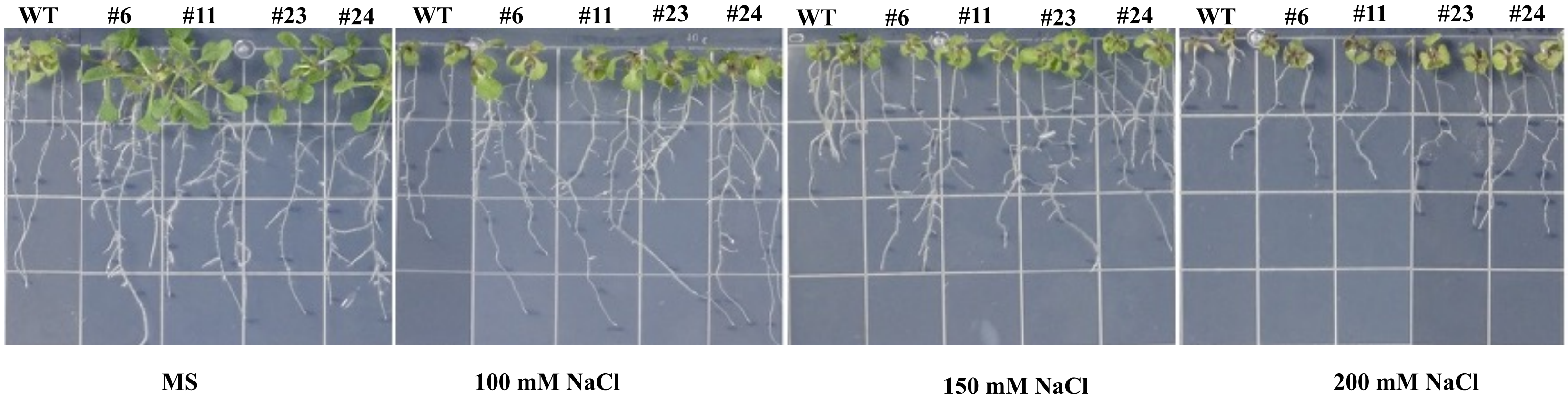
WT
#6
#11
#23
#24
WT
#6
#11
#23
#24
WT
#6
#11
#23
#24
WT
#6
#11
#23
#24
MS
100 mM NaCl
150 mM NaCl
200 mM NaCl

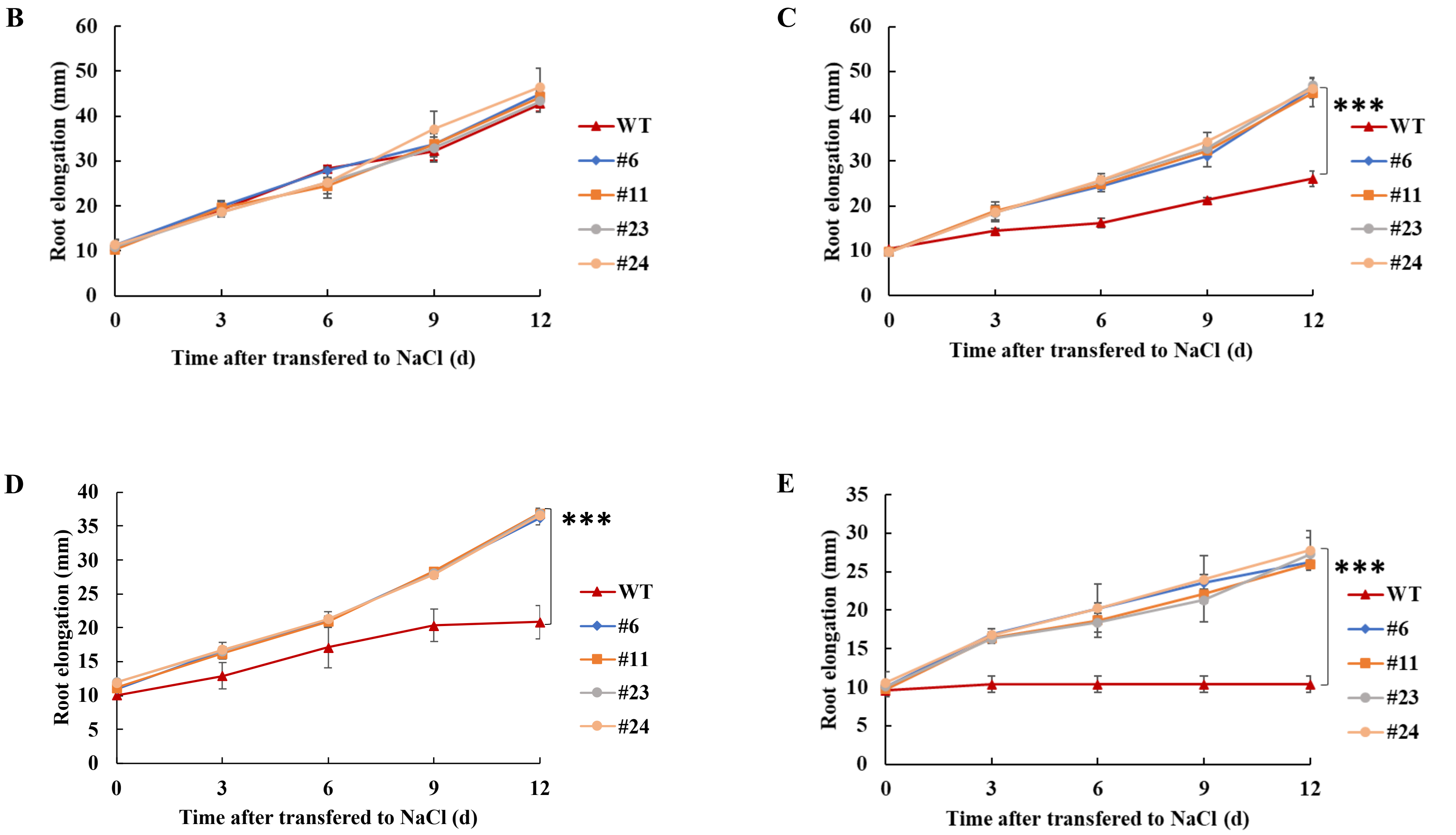
B
C
D
E
Root elongation (mm)
Time after transfered to NaCl (d)
WT
#6
#11
#23
#24
***

F

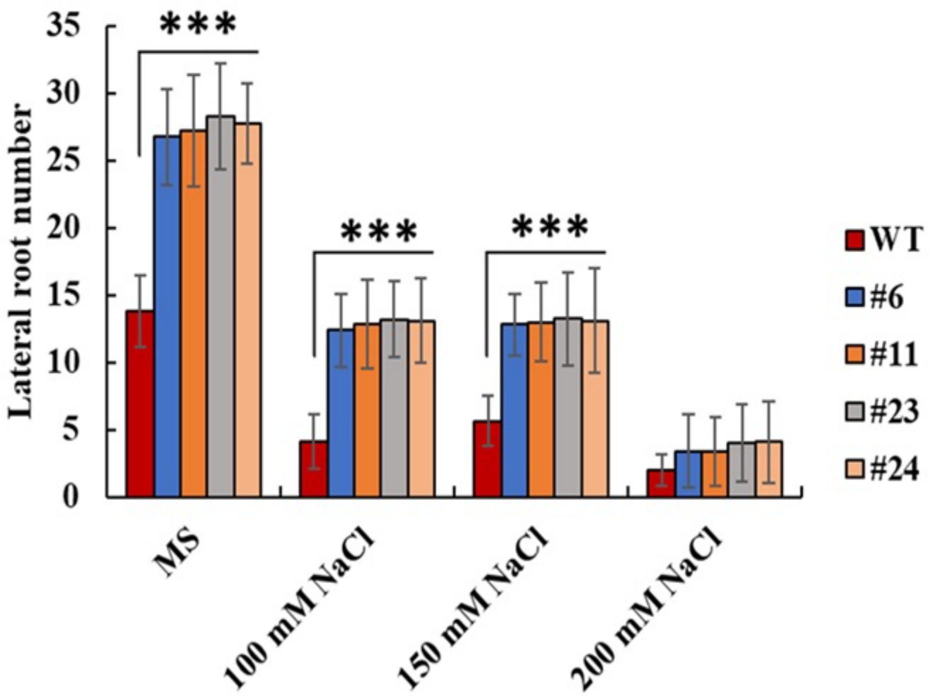

Figure S7. *KfNAC83* overexpression enhances root elongation and lateral root formation under NaCl stress. (**A**) Five-day-old seedlings were transferred to MS medium supplemented with 100, 150, or 200 mM NaCl and grown for 12 days. Root elongation was measured every 3 days. (**B–E**) Root elongation on MS, 100 mM, 150 mM, and 200 mM NaCl, respectively. (**F**) Number of lateral roots per plant after 12 days. Error bars represent ± SE from three independent experiments. ***$P < 0.001$. WT = wild type; #6, #11, #23, and #24 = independent transgenic lines. Scale bar = 1 cm.

A

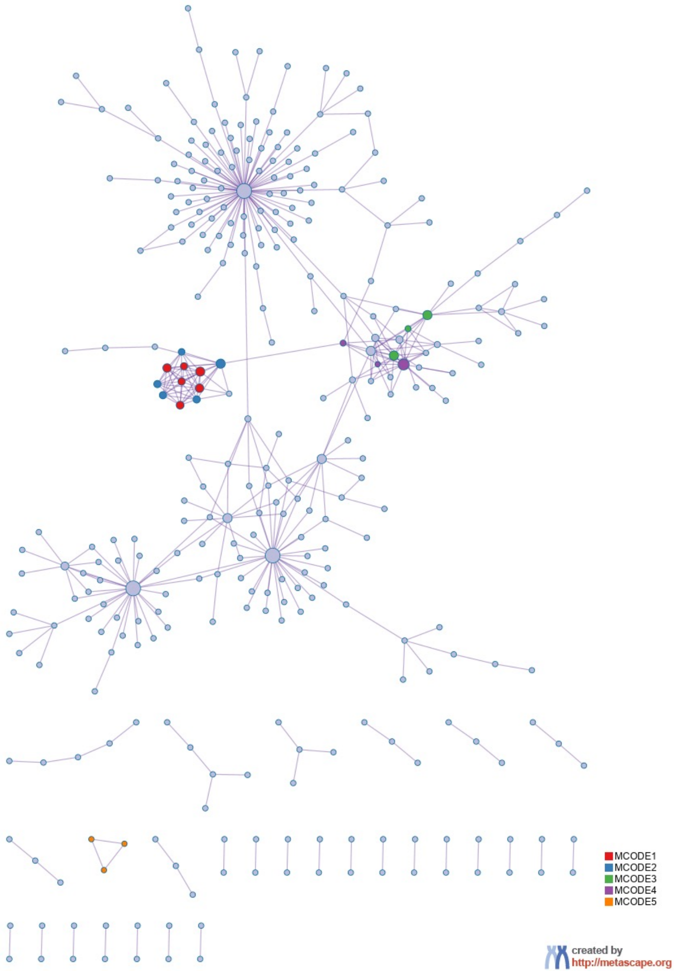
MCODE1
MCODE2
MCODE3
MCODE4
MCODE5
created by
http://metascape.org

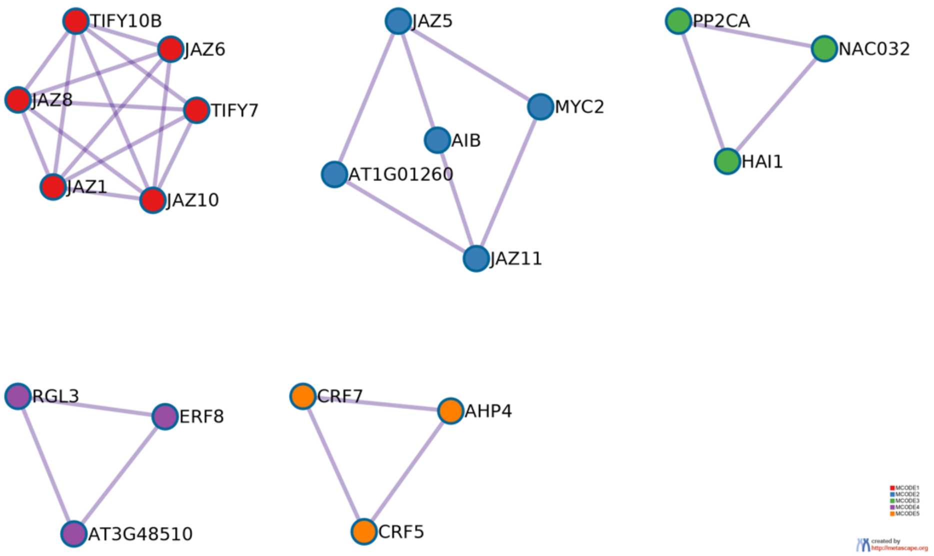
TIFY10B
JAZ6
JAZ8
TIFY7
JAZ1
JAZ10
JAZ5
MYC2
AIB
AT1G01260
JAZ11
PP2CA
NAC032
HAI1
RGL3
ERF8
AT3G48510
CRF7
AHP4
CRF5

B

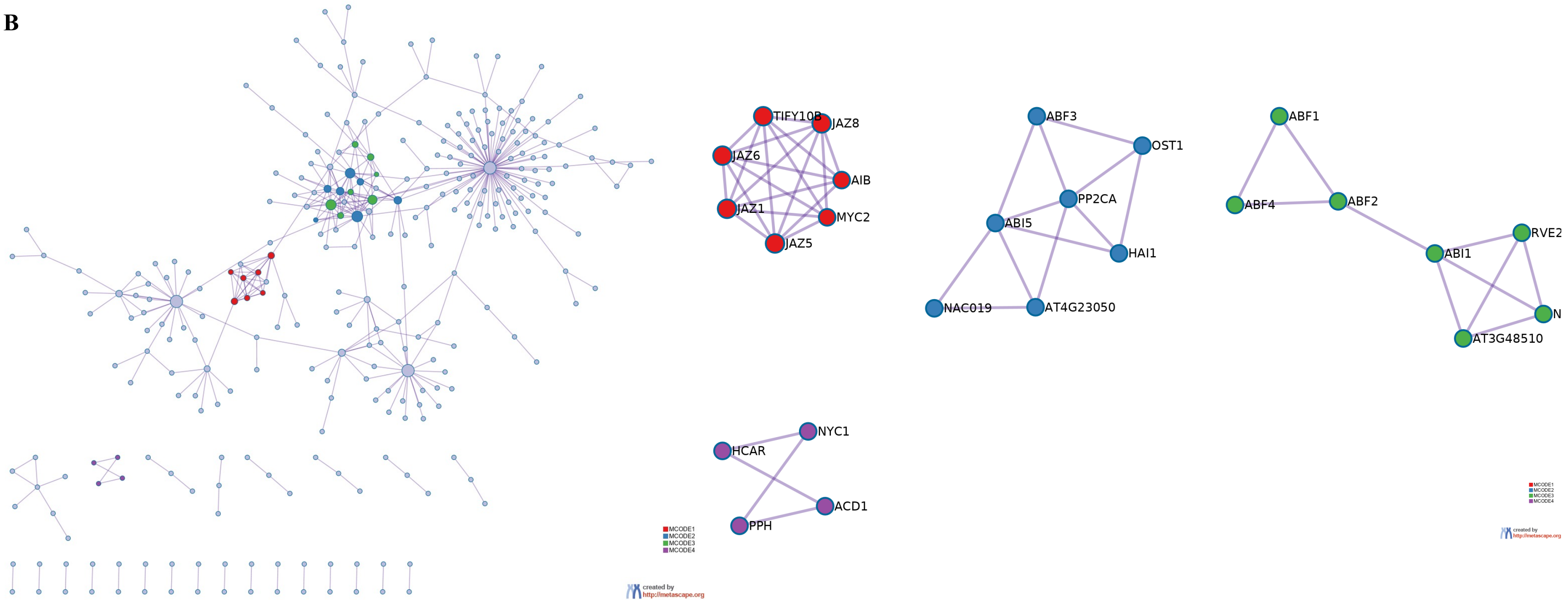
TIFY10B
JAZ8
JAZ6
AIB
JAZ1
MYC2
JAZ5
ABF3
OST1
PP2CA
ABI5
HAI1
NAC019
AT4G23050
ABF1
ABF4
ABF2
RVE2
ABI1
N
AT3G48510
NYC1
HCAR
ACD1
PPH
MCODE1
MCODE2
MCODE3
MCODE4
created by
http://metascape.org

C

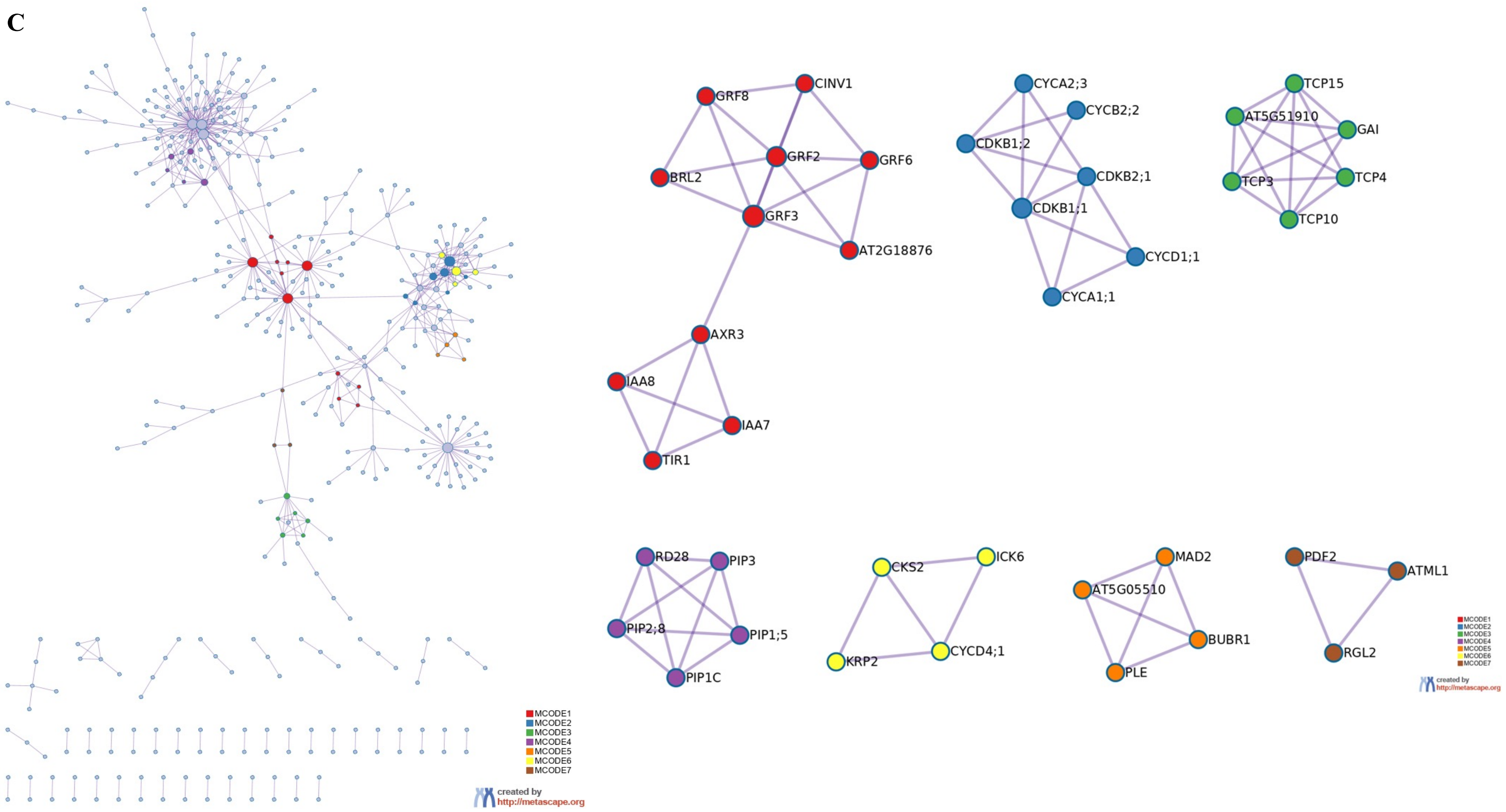

CINV1
GRF8
GRF2
GRF6
BRL2
GRF3
AT2G18876
AXR3
IAA8
IAA7
TIR1
CYCA2;3
CYCB2;2
CDKB1;2
CDKB2;1
CDKB1;1
CYCD1;1
CYCA1;1
TCP15
AT5G51910
GAI
TCP3
TCP4
TCP10
RD28
PIP3
PIP2;8
PIP1;5
PIP1C
CKS2
ICK6
KRP2
CYCD4;1
MAD2
AT5G05510
BUBR1
PLE
PDF2
ATML1
RGL2
MCODE1
MCODE2
MCODE3
MCODE4
MCODE5
MCODE6
MCODE7
created by
http://metascape.org

D

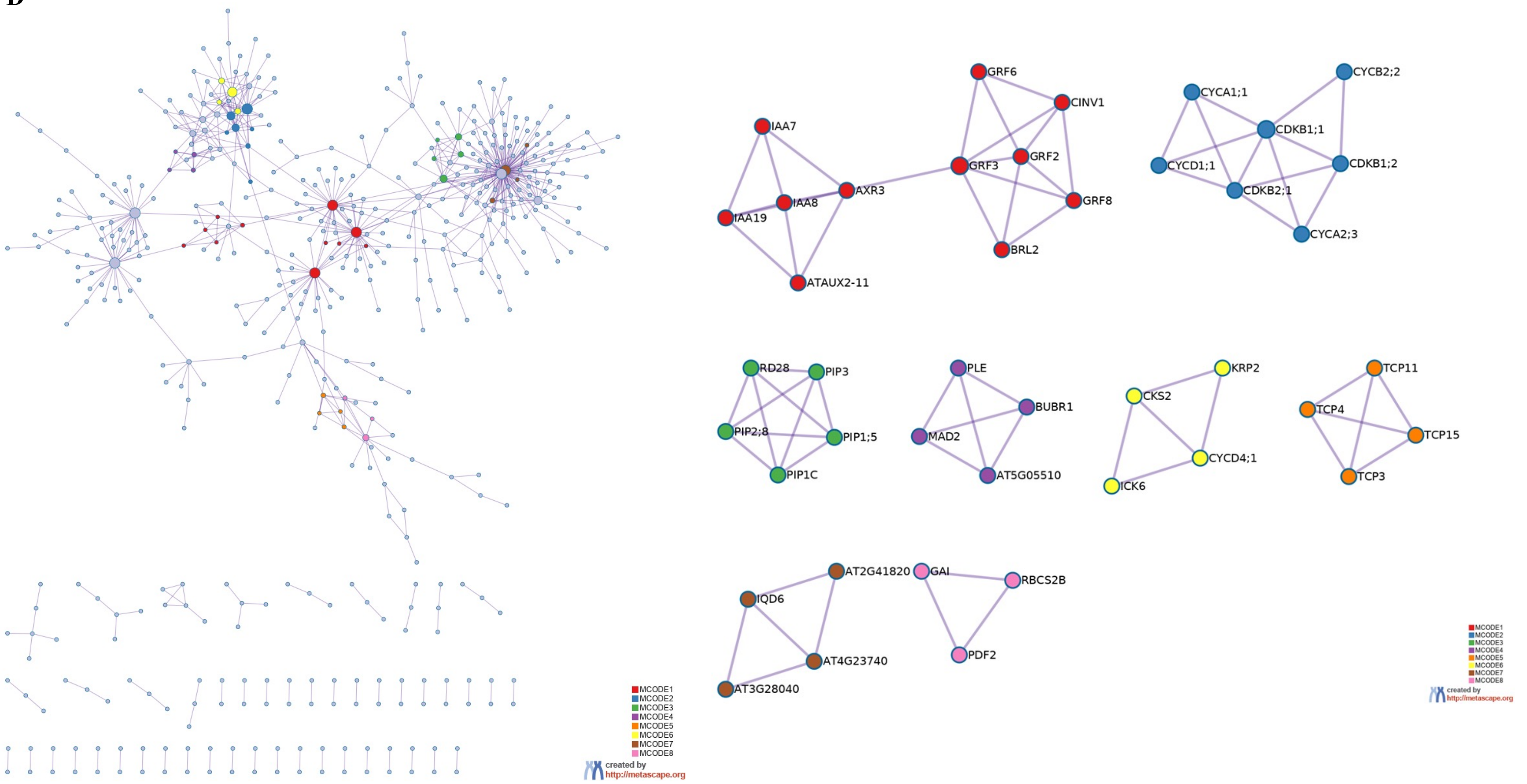

Figure S8. Protein–protein interaction (PPI) networks and MCODE modules of DEGs. PPI networks and MCODE-identified modules for (**A**) increased DEGs in *OxKfNAC83* line #24, (**B**) WT, (**C**) decreased DEGs in *OxKfNAC83*, and (**D**) WT. Nodes represent DEGs; edges represent predicted protein–protein interactions.

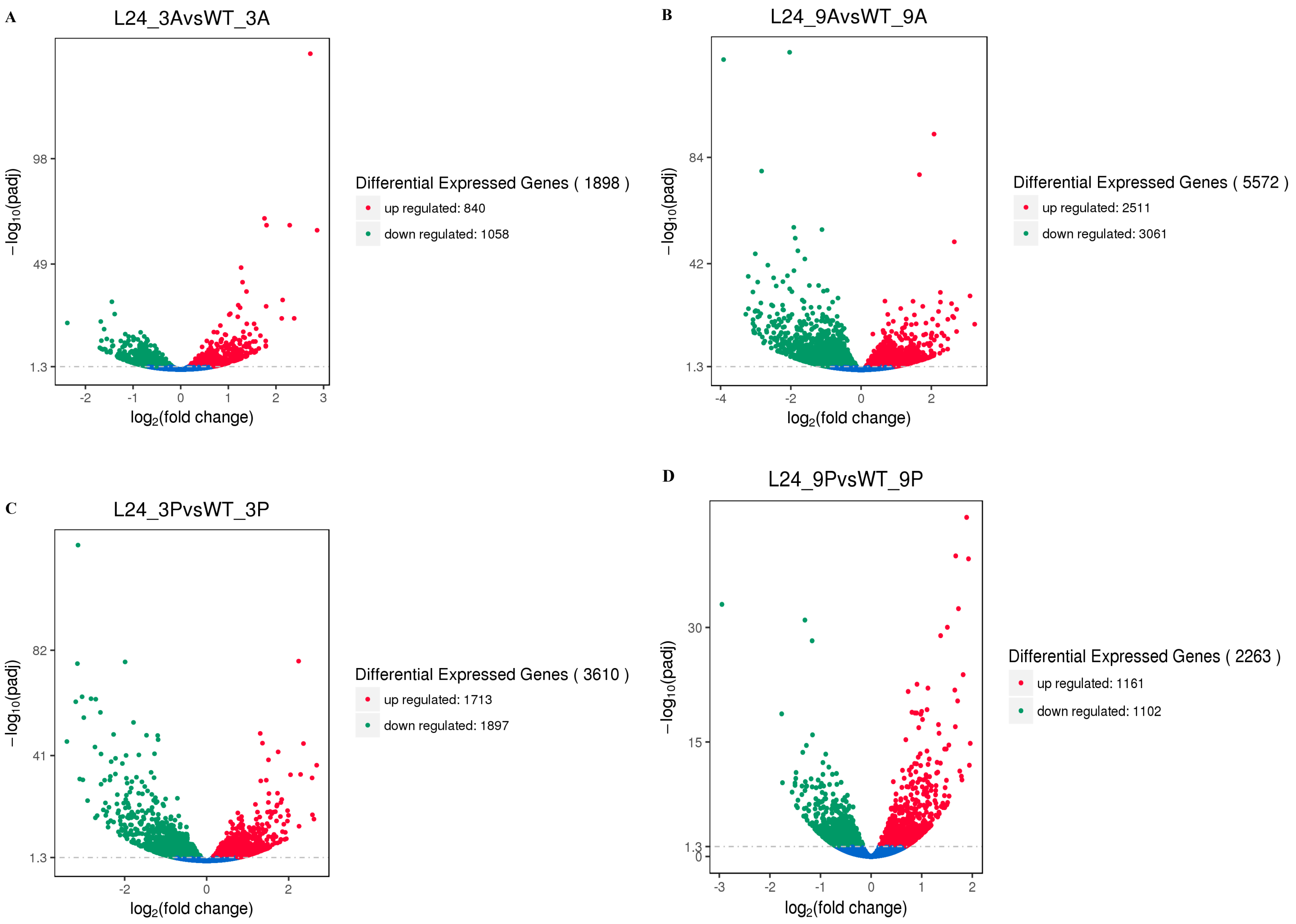
A
L24_3AvsWT_3A
−log10(padj)
98
49
1.3
-2
-1
0
1
2
3
log2(fold change)
Differential Expressed Genes (1898)
up regulated: 840
down regulated: 1058
B
L24_9AvsWT_9A
−log10(padj)
84
42
1.3
-4
-2
0
2
log2(fold change)
Differential Expressed Genes (5572)
up regulated: 2511
down regulated: 3061
C
L24_3PvsWT_3P
−log10(padj)
82
41
1.3
-2
0
2
log2(fold change)
Differential Expressed Genes (3610)
up regulated: 1713
down regulated: 1897
D
L24_9PvsWT_9P
−log10(padj)
30
15
1.3
0
-3
-2
-1
0
1
2
log2(fold change)
Differential Expressed Genes (2263)
up regulated: 1161
down regulated: 1102

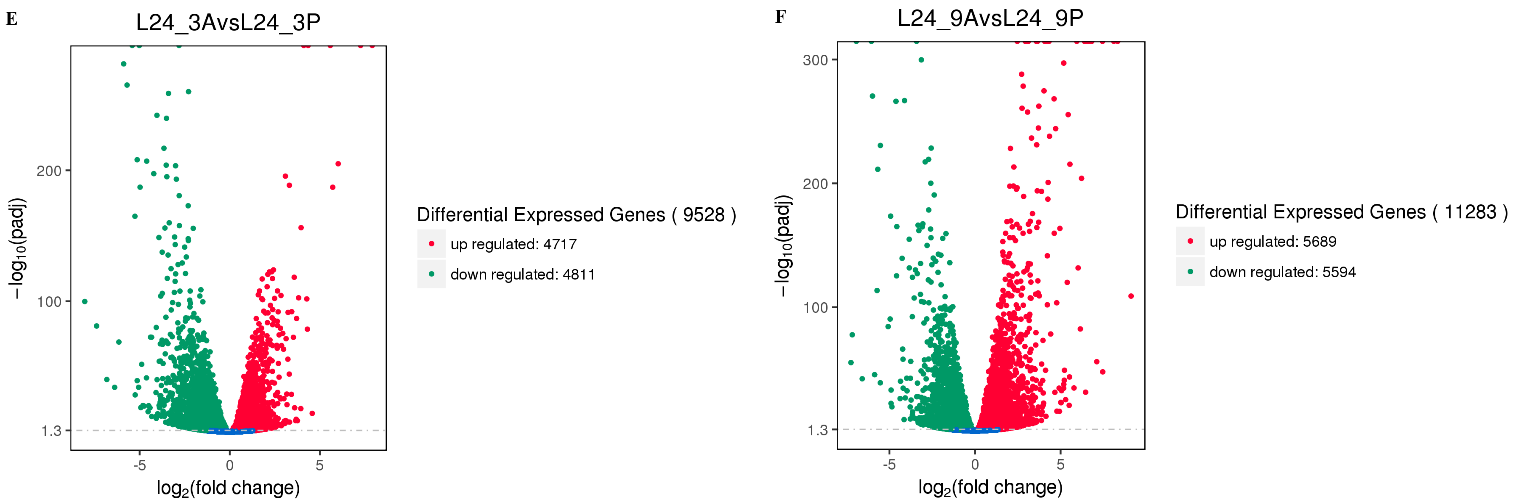

Figure S9. Volcano plots of differentially expressed genes (DEGs) across comparisons. Volcano plots show DEGs for (**A**) L24_3A vs WT_3A, (**B**) L24_9A vs WT_9A, (**C**) L24_3P vs WT_3P, (**D**) L24_9P vs WT_9P, (**E**) L24_3A vs L24_3P, and (**F**) L24_9A vs L24_9P. The y-axis represents statistical significance ($-\log_{10}$ adjusted P-value); the x-axis represents $\log_2$ fold change. Red = decreased, green = increased, blue = not significant.

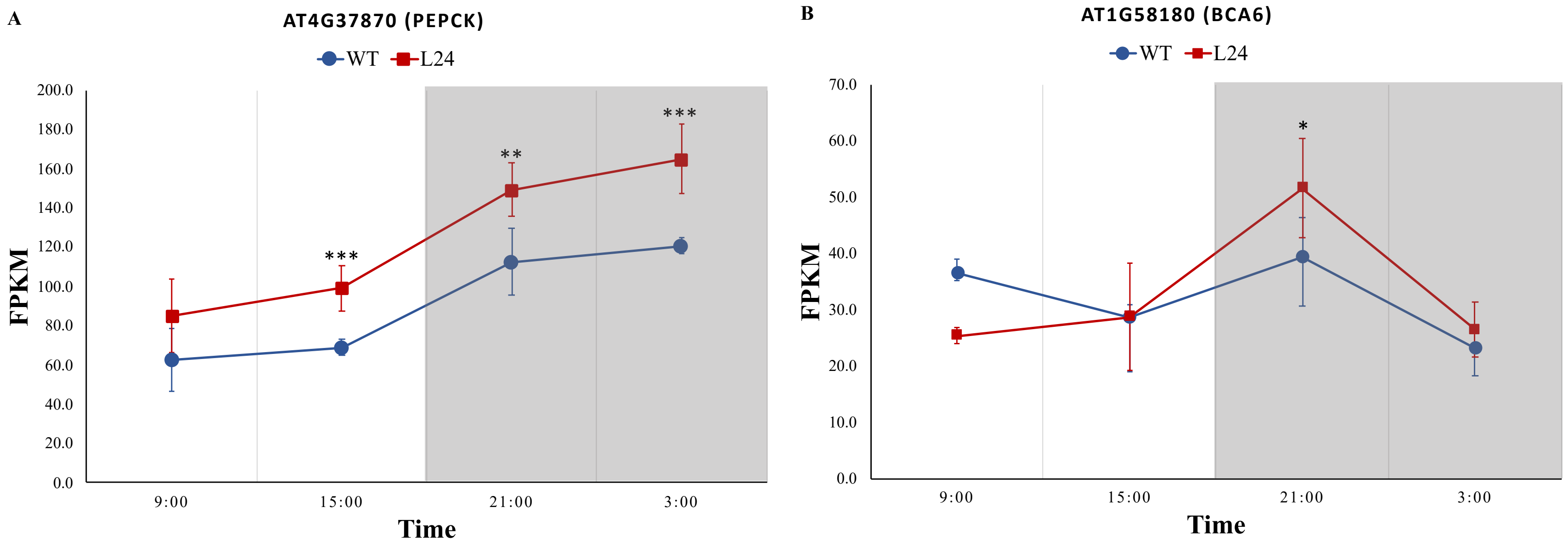

A
AT4G37870 (PEPCK)
WT
L24
200.0
180.0
160.0
140.0
120.0
100.0
80.0
60.0
40.0
20.0
0.0
FPKM
***
**
***
9:00
15:00
21:00
3:00
Time
B
AT1G58180 (BCA6)
WT
L24
70.0
60.0
50.0
40.0
30.0
20.0
10.0
0.0
FPKM
*
9:00
15:00
21:00
3:00
Time

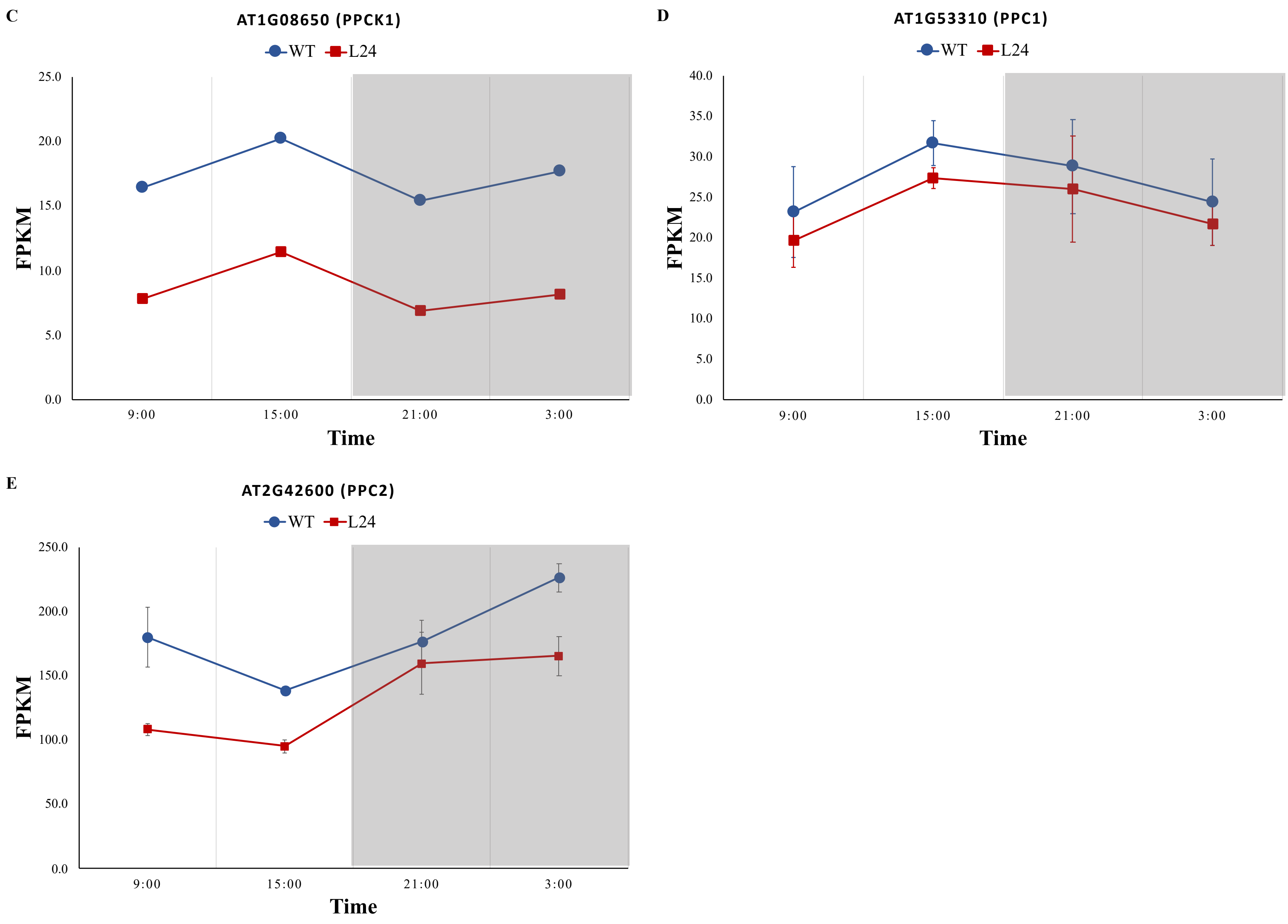
C
AT1G08650 (PPCK1)
WT
L24
25.0
20.0
15.0
10.0
5.0
0.0
FPKM
9:00
15:00
21:00
3:00
Time
D
AT1G53310 (PPC1)
WT
L24
40.0
35.0
30.0
25.0
20.0
15.0
10.0
5.0
0.0
FPKM
9:00
15:00
21:00
3:00
Time
E
AT2G42600 (PPC2)
WT
L24
250.0
200.0
150.0
100.0
50.0
0.0
FPKM
9:00
15:00
21:00
3:00
Time

Figure S10. Expression of core CAM-related genes over a 24-hour period. FPKM (fragments per kilobase of exon per million mapped reads) values show diel expression patterns of CAM-related genes in *OxKfNAC83* and WT plants. (**A**) PEPCK. (**B**) BCA6. (**C**) PPCK1. (**D**) PPC1. (**E**) PPC2

A

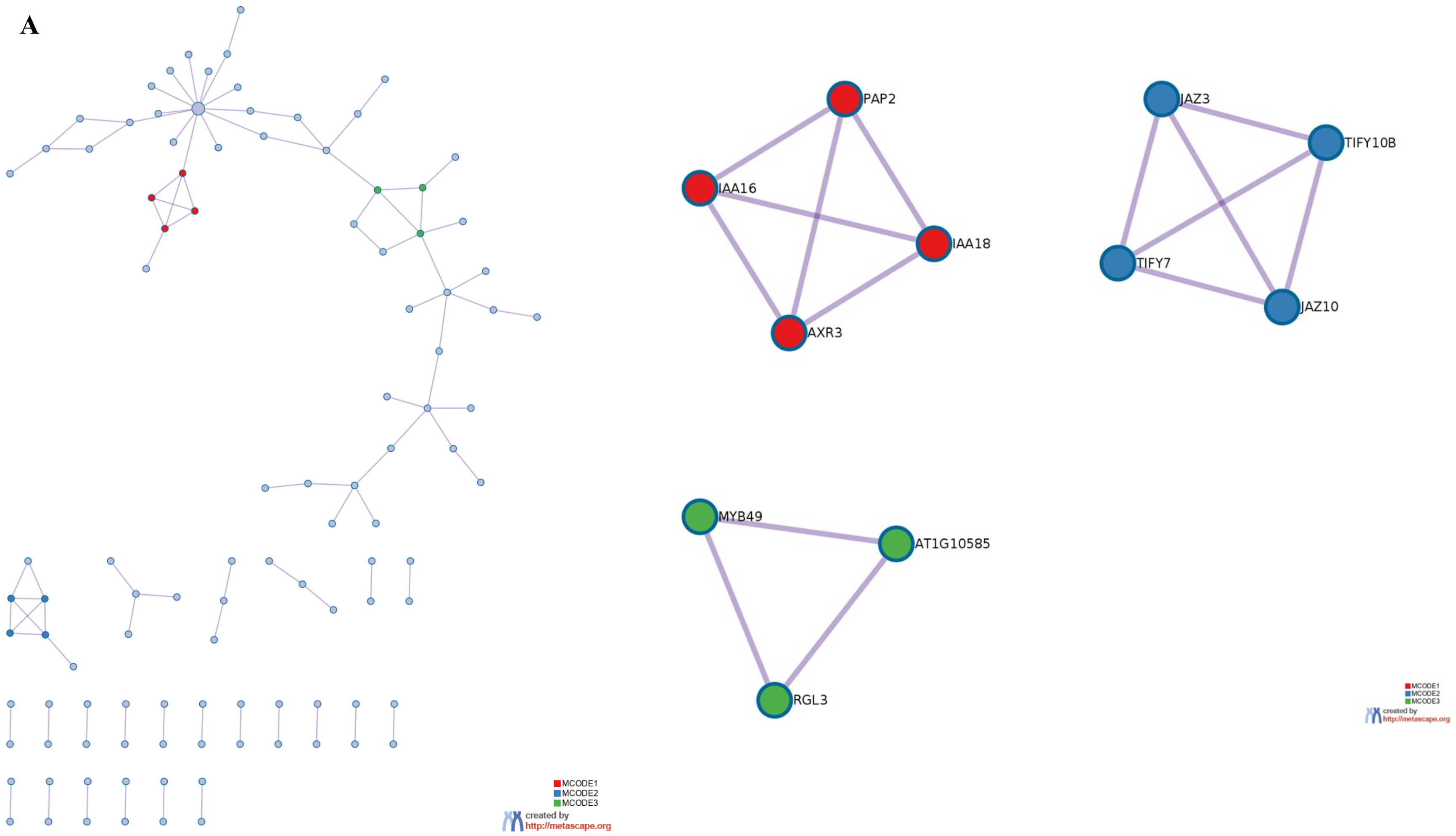
MCODE1
MCODE2
MCODE3
created by
http://metascape.org
PAP2
IAA16
IAA18
AXR3
JAZ3
TIFY10B
TIFY7
JAZ10
MYB49
AT1G10585
RGL3
MCODE1
MCODE2
MCODE3
created by
http://metascape.org

B

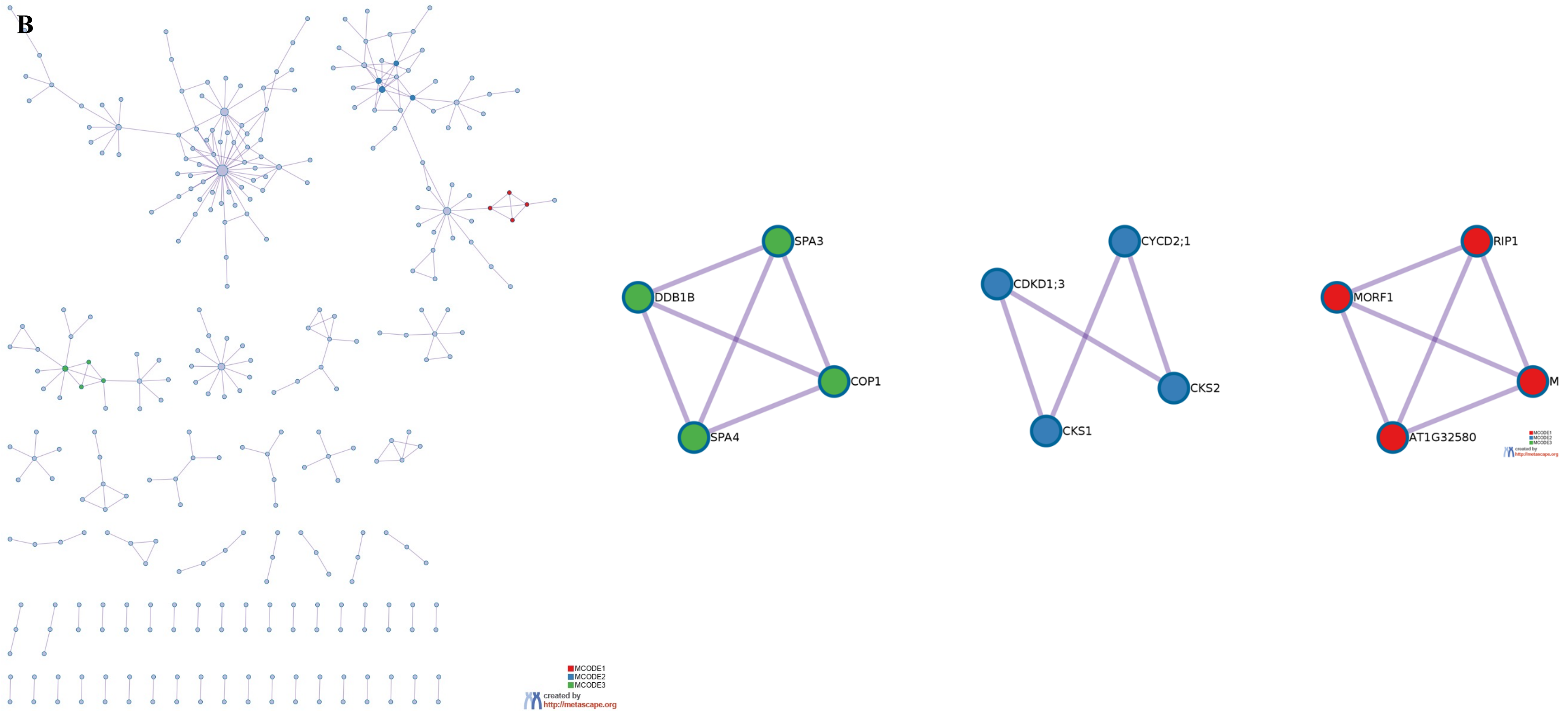
MCODE1
MCODE2
MCODE3
created by
http://metascape.org
SPA3
DDB1B
COP1
SPA4
CYCD2;1
CDKD1;3
CKS2
CKS1
RIP1
MORF1
M
AT1G32580

C

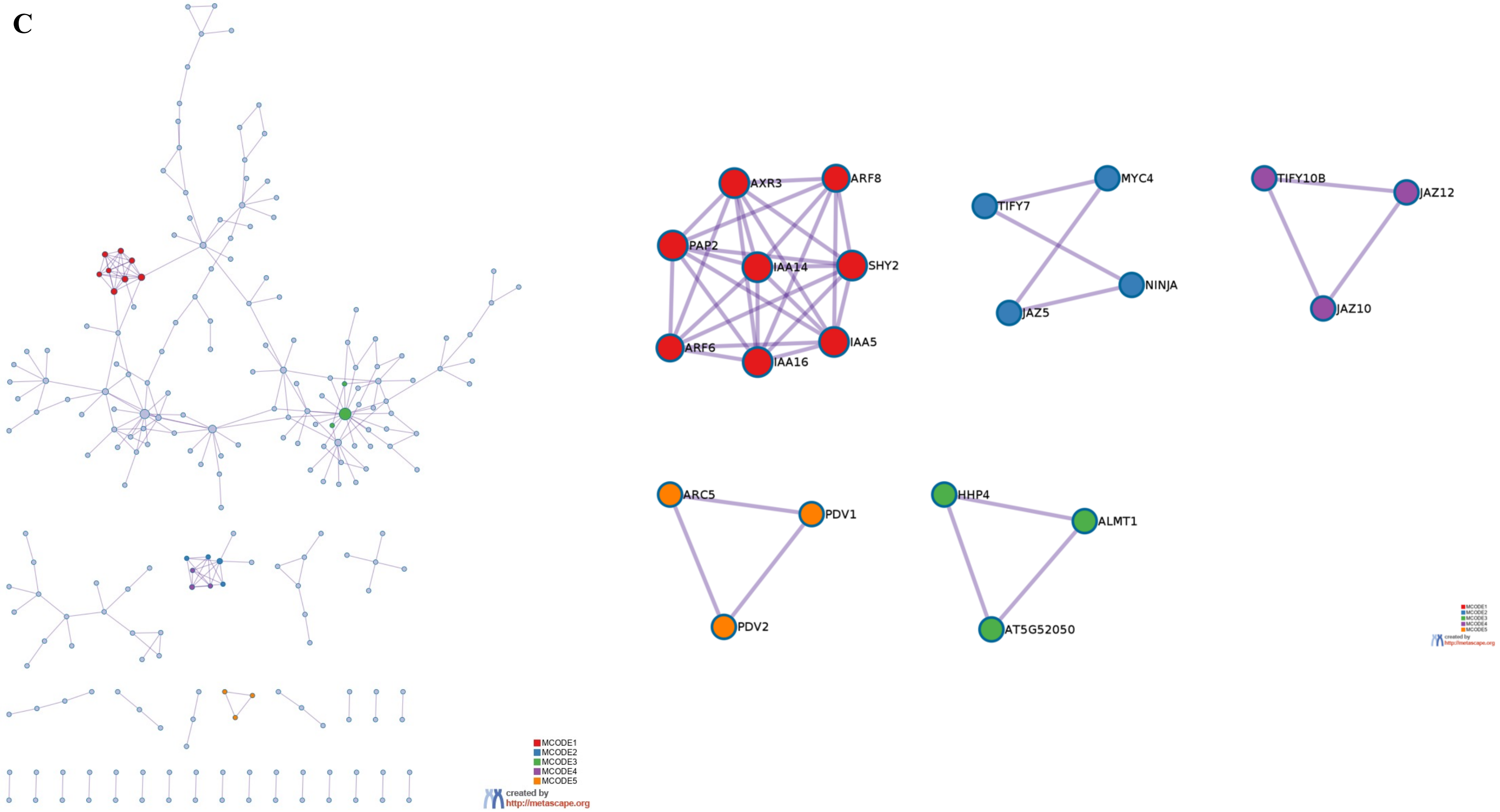
AXR3
ARF8
PAP2
IAA14
SHY2
ARF6
IAA16
IAA5
MYC4
TIFY7
NINJA
JAZ5
TIFY10B
JAZ12
JAZ10
ARC5
PDV1
PDV2
HHP4
ALMT1
AT5G52050
MCODE1
MCODE2
MCODE3
MCODE4
MCODE5
created by
http://metascape.org

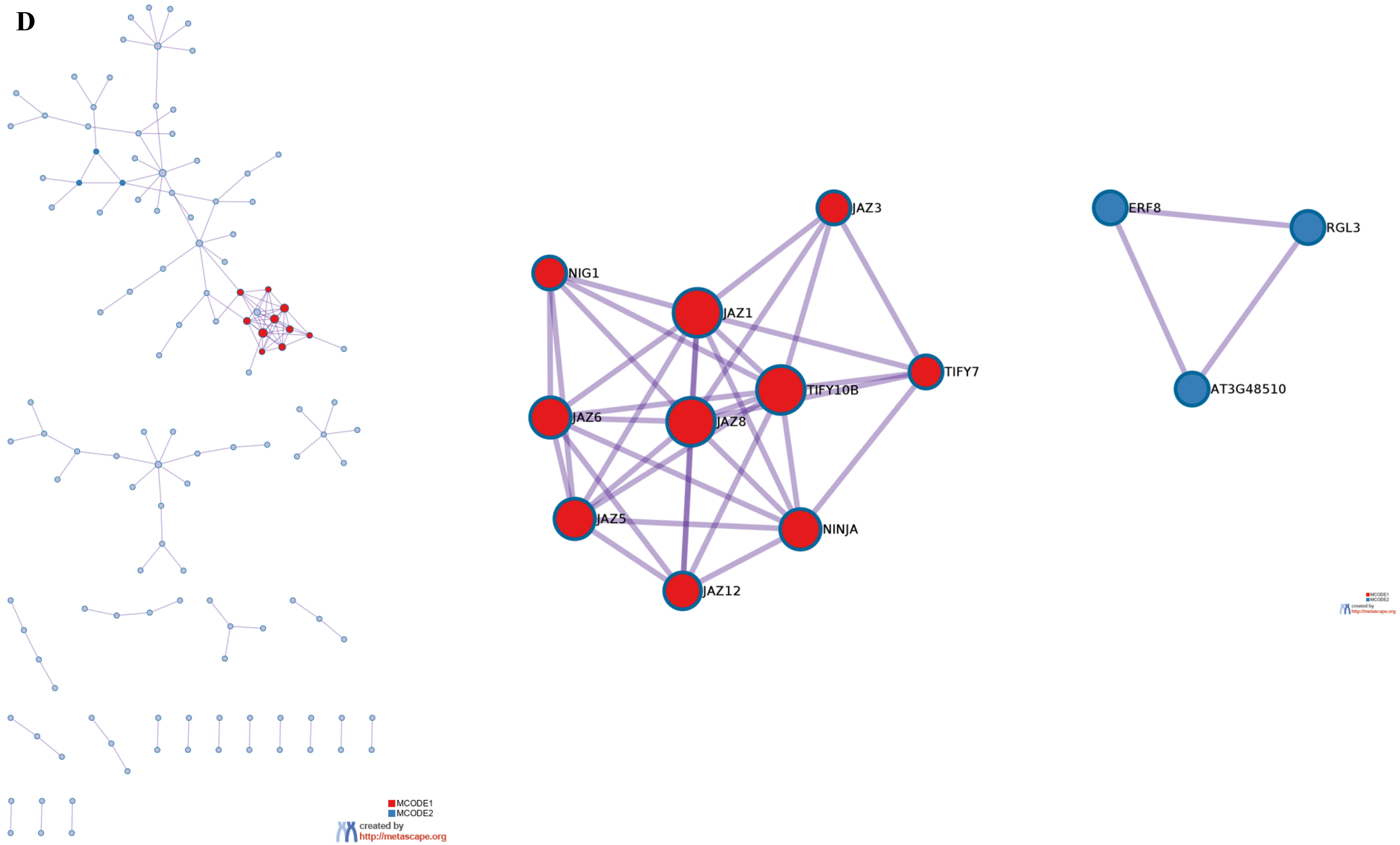

Figure S11. PPI networks and MCODE modules of upregulated DEGs across time points. PPI networks and MCODE modules for increased DEGs in (A) L24_3A vs WT_3A, (**B**) L24_9A vs WT_9A, (**C**) L24_3P vs WT_3P, and (**D**) L24_9P vs WT_9P. Nodes represent decreased DEGs; edges represent predicted interactions.
